# A systematic analysis of hypermucoviscosity and capsule reveals distinct and overlapping genes that impact *Klebsiella pneumoniae* fitness

**DOI:** 10.1371/journal.ppat.1009376

**Published:** 2021-03-15

**Authors:** Laura A. Mike, Andrew J. Stark, Valerie S. Forsyth, Jay Vornhagen, Sara N. Smith, Michael A. Bachman, Harry L. T. Mobley

**Affiliations:** 1 Department of Microbiology & Immunology, University of Michigan, Ann Arbor, Michigan, United States of America; 2 Department of Pathology, University of Michigan, Ann Arbor, Michigan, United States of America; Tufts University, UNITED STATES

## Abstract

Hypervirulent *K*. *pneumoniae* (hvKp) is a distinct pathotype that causes invasive community-acquired infections in healthy individuals. Hypermucoviscosity (hmv) is a major phenotype associated with hvKp characterized by copious capsule production and poor sedimentation. Dissecting the individual functions of CPS production and hmv in hvKp has been hindered by the conflation of these two properties. Although hmv requires capsular polysaccharide (CPS) biosynthesis, other cellular factors may also be required and some fitness phenotypes ascribed to CPS may be distinctly attributed to hmv. To address this challenge, we systematically identified genes that impact capsule and hmv. We generated a condensed, ordered transposon library in hypervirulent strain KPPR1, then evaluated the CPS production and hmv phenotypes of the 3,733 transposon mutants, representing 72% of all open reading frames in the genome. We employed forward and reverse genetic screens to evaluate effects of novel and known genes on CPS biosynthesis and hmv. These screens expand our understanding of core genes that coordinate CPS biosynthesis and hmv, as well as identify central metabolism genes that distinctly impact CPS biosynthesis or hmv, specifically those related to purine metabolism, pyruvate metabolism and the TCA cycle. Six representative mutants, with varying effect on CPS biosynthesis and hmv, were evaluated for their impact on CPS thickness, serum resistance, host cell association, and fitness in a murine model of disseminating pneumonia. Altogether, these data demonstrate that hmv requires both CPS biosynthesis and other cellular factors, and that hmv and CPS may serve distinct functions during pathogenesis. The integration of hmv and CPS to the metabolic status of the cell suggests that hvKp may require certain nutrients to specifically cause deep tissue infections.

## Author summary

*Klebsiella pneumoniae* is a common multi-drug resistant hospital-associated pathogen, however some isolates are capable of causing community-acquired infections in otherwise healthy individuals. The strains causing community-acquired infections have some distinguishing characteristics, which include overproduction of capsule and hypermucoviscosity. Hypermucoviscous strains are very tacky and sediment poorly when centrifuged. Historically, hypermucoviscosity has been attributed to overproduction of capsular polysaccharide, but recent data suggest that other factors contribute to this bacterial phenotype. Moreover, it seems that capsule and hypermucoviscosity may have distinct roles in pathogenesis. In this study, we sought to systematically investigate the genes that contribute to capsule and hypermucoviscosity. We found that in most cases, genes coordinately impact both capsule biosynthesis and hypermucoviscosity. Some metabolic genes linked to the TCA cycle, however, only affect one of these properties. Here, we identify that capsule biosynthesis and hypermucoviscosity are tightly tied to central metabolism and that an optimal balance between metabolism, capsule, and hypermucoviscosity are important for *in vivo* fitness of *K*. *pneumoniae*. These results identify genes that can be further probed to dissect how capsule and hypermucoviscosity are coordinated in response to niche-specific nutrients. Such studies will expand our understanding of the factors that drive the pathobiology of hypervirulent *K*. *pneumoniae*.

## Introduction

*Klebsiella pneumoniae* is a ubiquitous bacterium found in a range of environments, including soil, sewage, sink P-traps, and mammalian gastrointestinal tracts. Colonization of the human gut with *K*. *pneumoniae* is a risk factor for infection, which commonly manifests as hospital-associated pneumonia, urinary tract infections (UTI), and bacteremia [1–3]. Classical *K*. *pneumoniae* (cKp) is commonly an opportunistic pathogen causing infections in patients who are immunocompromised, have indwelling medical devices, have undergone an invasive medical procedure, or have other co-morbidities such as diabetes mellitus and alcoholism [4,5]. With human colonization rates reported at 23–36%, increasing antibiotic resistance, and a non-fastidious lifestyle, it is not surprising that *K*. *pneumoniae* is the third most common nosocomial pathogen [1,3,4,6].

Two clinically challenging pathotypes with high morbidity and mortality are the carbapenem-resistant, classical *K*. *pneumoniae* (CR-cKp) and hypervirulent *K*. *pneumoniae* (hvKp) [5,7–9]. CR-cKp was first observed in 1996 and since then has been the major driving force disseminating carbapenem-resistance throughout the Enterobacteriaceae, complicating the treatment of many gram-negative infections [10,11]. In parallel, hvKp incidence is rising in both community and hospital settings [12–15]. While hvKp is susceptible to most antibiotics, it is associated with invasive infections in otherwise healthy patients and is notorious for causing pyogenic liver abscesses and disseminating to the eyes, lungs and brain, a pathogenesis uncommon for gram-negative enteric bacteria [3,7,8]. HvKp mortality rates range from 3 to 55% and survivors often have severe morbidities such as vision loss or neurologic sequelae [7,8]. Alarmingly, the CR-cKp and hvKp pathotypes can converge [3,16]. The prevalence of CR-hvKp is 7.4–15% in countries where hvKp is endemic, demonstrating that more devastating *K*. *pneumoniae* lineages are emerging [7].

**Fig 1:**
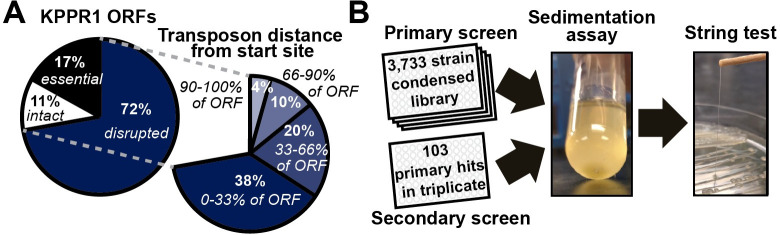
A forward phenotypic screen of the condensed, ordered *K*. *pneumoniae* library. (A) A Mariner/*Himar1* transposon (Tn) library was generated in *K*. *pneumoniae* strain KPPR1 and arrayed into 192, 96-well microplates. 14,895 traceable Tn insertions were mapped, disrupting 71.6% of open reading frames (ORFs) and 74.2% of predicted transcriptional units. The best representative Tn insertion for the 3,733 disrupted genes were consolidated into a condensed library. 58% of all Tn insertion sites are located within the first 66.7% of each ORF. (B) The condensed transposon library was then screened for non-mucoviscous mutants as indicated by improved sedimentation (microplate, 2,000 x *g* for 20 min). Strains with supernatant OD_600_ values two standard deviations below the plate mean were then evaluated on solid medium by string test. Each hit was arrayed in triplicate into microplates and screened a second time for hypermucoviscosity by sedimentation (2,000 x *g* for 20 min) and string test.

Accessory features associated with hvKp include hypermucoviscosity (hmv), K1 or K2 capsule-types, overexpression of RmpA (regulator of mucoid phenotype), and stealth siderophore biosynthesis [7,12,14,15,17,18]. Traditionally, *K*. *pneumoniae* isolates are categorized as hmv by string test if their colony stretches more than five mm when picked off a plate (**Fig 1B**). In addition, overexpression of RmpA has been shown to increase capsular polysaccharide (CPS) production [19,20]. A clear link between CPS and virulence has been demonstrated in multiple murine models of *K*. *pneumoniae* infection, including pneumonia and UTI [21,22]. Despite CPS being a key fitness factor for *K*. *pneumoniae*, the regulatory network that directly controls CPS biosynthesis is not fully understood. A recent study reported the *K*. *pneumoniae* CPS biosynthesis regulatory network using density-TraDISort [23]. Transposon mutant pools were separated over a discontinuous Percoll gradient to separate populations with altered buoyancy as a surrogate measure of CPS production in two hvKp strains, NTUH-K2044 and ATCC 43816 [23]. Transposon insertions were identified that increase buoyancy in NTUH-K2044 or decrease buoyancy in NTUH-K2044 and/or ATCC 43816, then the hmv and CPS production of ten targeted deletion mutants were quantified to validate the density-TraDISort. Building upon this study, we sought to systematically expand our understanding of the relationship between CPS biosynthesis and hmv using all available genes of interest identified by density-TraDISort.

Historically, hmv has been closely associated with hvKp and attributed to over-production of CPS, as hmv is lost in the absence of CPS biosynthesis [7]. This paradigm is pervasive throughout the *K*. *pneumoniae* literature despite early studies suggesting that hmv may not only be due to overproduction of CPS [24,25]. More recently, discordant changes in CPS production and hmv have been shown at the phenotypic and genotypic levels [3,26,27]. Some examples include the *rmpC* mutant in strain KPPR1S, which exhibits reduced CPS production, but retains full hmv; and the *rmpD* mutant in strain KPPR1S, which synthesizes WT levels of CPS, but is non-mucoviscous [26,27]. In fact, recent data have shown that *rmpA*, *rmpD*, and *rmpC* form a single operon, where RmpA auto-regulates the operon, *rmpD* expression increases hmv independently of capsule biosynthesis, and *rmpC* expression increases capsule biosynthesis without impacting hmv [27]. The mounting evidence that hmv and CPS overproduction has been conflated into a single characteristic of hvKp spotlights our limited understanding of *K*. *pneumoniae* hypervirulence and points to the critical need to better understand the relationship and function of these two important features in *K*. *pneumoniae* pathogenesis and biology.

To systematically evaluate the relationship between CPS biosynthesis and hmv and provide a robust resource for future molecular studies, we have developed an ordered transposon library in the hypervirulent *K*. *pneumoniae* strain KPPR1 using Cartesian-Pooling and Coordinate-Sequencing [28]. We then condensed the library to include a representative mutant for each of the 3,733 disrupted genes. To validate the use of the library and more broadly examine the relationship between hmv and CPS overproduction, forward and reverse genetic screens were performed to systematically quantify both CPS production and hmv exhibited by transposon mutants. The use of a forward screen allowed for the unbiased identification of mutants that impact hmv and/or CPS production, while the reverse screen enabled methodical screening of CPS production and hmv for genes previously ascribed to affect *K*. *pneumoniae* buoyancy [23]. Global analyses of the CPS production and hmv of 100 transposon mutants and 27 targeted deletion mutants revealed a significant correlation between the two biological features, although several mutants displayed discordant regulation of hmv and CPS biosynthesis. These data strengthen the emerging model that CPS production and hmv are tightly linked, but distinct; emphasizing the need to decouple these features and define their individual contributions to hypervirulence. Examination of six representative mutants with a variety of hmv and CPS phenotypes by TEM revealed that CPS thickness does not correlate with hmv; however, amongst these same strains, those with reduced CPS production were more sensitive to human serum, while those with reduced hmv associated more tightly with human lung epithelial cells. Since hvKp typically cause invasive infections that exhibit metastatic spread [5], we used a murine model of disseminating pneumonia to evaluate the *in vivo* fitness of these six representative mutants. All were significantly out-competed *in vivo*, suggesting that coordinated regulation of CPS biosynthesis and hmv are critical for maximal fitness. Therefore, it is of utmost importance that the overlapping and distinct pathways controlling hmv and CPS biosynthesis be further mapped so that the functional relationships between hmv, CPS, and hypervirulence in *K*. *pneumoniae* can be further dissected. Such studies may ultimately identify targets useful for specifically treating hvKp infections.

## Results

### Generating a condensed, ordered transposon library in *Klebsiella pneumoniae* strain KPPR1

To facilitate both forward and reverse genetic studies in *K*. *pneumoniae*, an ordered transposon library of strain KPPR1, a rifampin-resistant derivative of ATCC 43816 that has a K2 capsule type and is hypermucoviscous, was generated [29]. Mariner *Himar1* transposon mutants were arrayed into 192, 96-well microplates and the transposon insertion site present in each well of the library was identified using Cartesian Pooling and Coordinate Sequencing (CP-CSeq) [28]. The library contains 14,895 traceable transposon mutants that disrupt 3,733 genes, covering 71.6% and 74.2% of predicted open reading frames (ORFs) and transcriptional units, respectively (**Table 1** and **S1 Data**). For each gene disrupted, a representative transposon mutant was selected to generate a condensed library (**S1 Data**). The representative mutant for each ORF was selected based on the confidence of its unique positional location in the library and the proximity of the transposon insertion site to the translational start site; 58% of all transposon insertion sites are in the first 66.7% of the ORF (**Fig 1A**). The selected mutants, representing 87% of KPPR1 non-essential genes, were arrayed into 41 microplates (**Fig 1A** and **S1 Data**) [30]. The accuracy of CP-CSeq identification of mutant positional locations was evaluated by PCR, where 92.9% (N = 14) and 93.8% (N = 16) of tested transposon mutants from the complete and condensed libraries, respectively, were confirmed to have the expected transposon insertion site. It is important to recognize that (1) absence of PCR product does not preclude the possibility that the correct transposon insertion site was present, but not detected by the PCR, (2) presence of PCR product does not exclude the possibility of additional transposon insertion sites sharing the library location, and (3) one mutant that did not validate in the condensed library grew poorly.

**Table 1:** Composition of ordered and condensed *K*. *pneumoniae* strain KPPR1 transposon libraries.

	ORDERED LIBRARY	CONDENSED LIBRARY
**Total number of insertions**	18,598	3,733
**Intergenic**	2,820	0
**Single open reading frame (ORF)**	12,035	3,715
**Unique location**	10,641	3,605
**Plate-OK unique^a^**	413	46
**Plate-OK non-unique^b^**	25	2
**Well-OK unique^c^**	458	38
**Well-OK non-unique^d^**	30	3
**Heuristic^e^**	468	21
**Two contiguous ORFs^f^**	40	18
**Non-traceable^g^**	3,703	0
**>1 insertion site^h^**	2,062	216

^a^Plate location mapped as expected, well location reads mapped to the most probable position

^b^Plate location mapped as expected, multiple well position possible

^c^Well location mapped as expected, plate location reads mapped to the most probable position

^d^Well location mapped as expected, multiple plate position possible

^e^Multiple plate and well locations are possible

^f^Insertion site was mapped to a location where two ORFs overlap

^g^Reads were mapped to the genome, but a location could not be assigned

^h^More than 1 transposon insertion site mapped to a single well

### An unbiased forward phenotypic screen identified genes that influence hypermucoviscosity and capsular polysaccharide production

The classification of an isolate as hmv is typically done by a string test, where a colony is lifted off a plate with an inoculating loop and if it stretches more than five mm it is considered hmv (**Fig 1B**). *K*. *pneumoniae* hmv can also be quantified in liquid cultures by sedimentation since hypermucoviscous cells are retained in the supernatant after centrifugation, while non-mucoviscous cells fully sediment to form a tight pellet [27,31]. This objective assay is more quantitative and reproducible than the string test.

**Fig 2:**
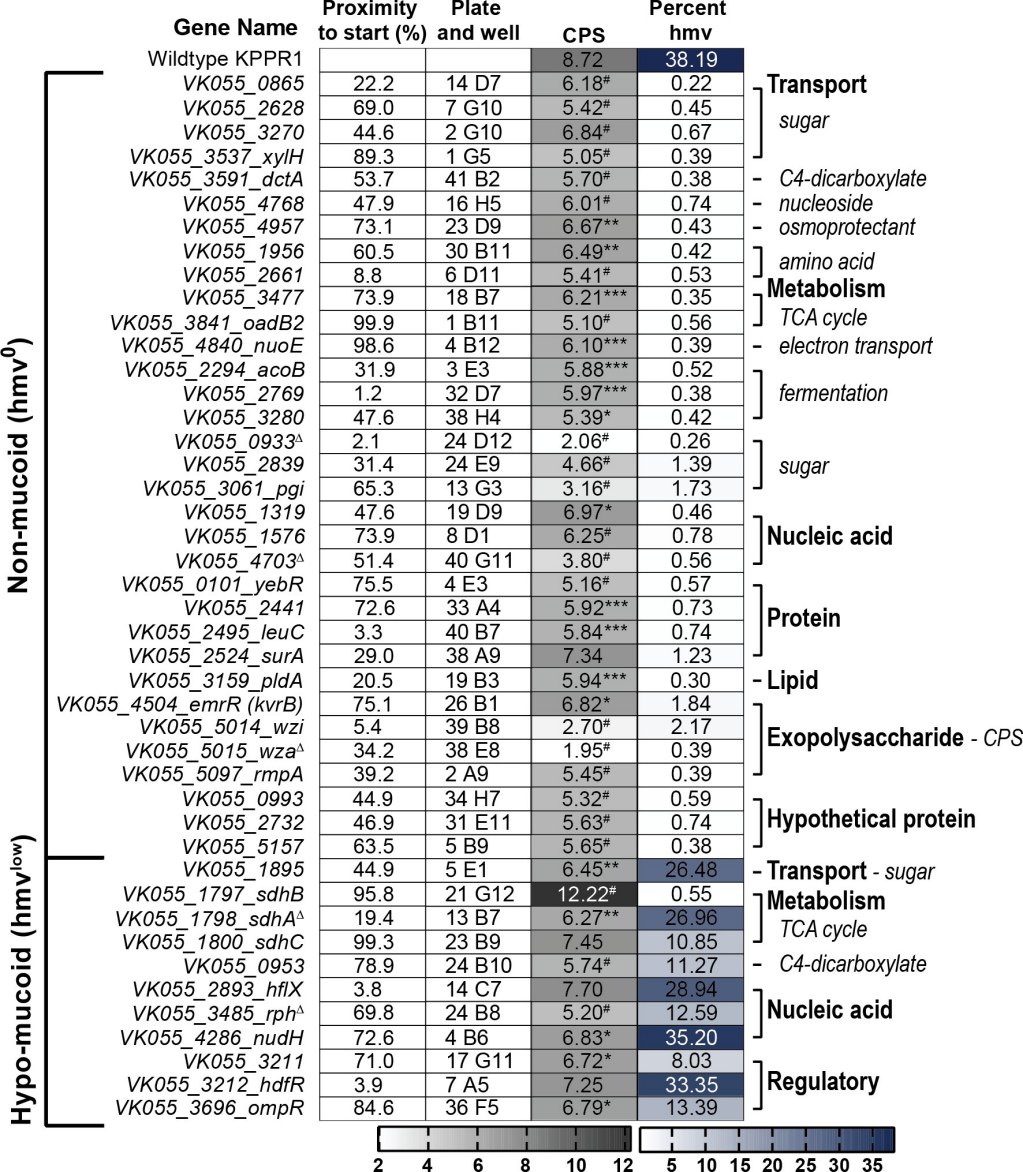
Forward phenotypic screen of KPPR1 hypermucoviscosity and capsular polysaccharide biosynthesis. Shown are the results of the forward screen identifying genes that influence hypermucoviscosity (hmv) and/or capsular polysaccharide (CPS) biosynthesis, where the predicted gene function is annotated in the right margin. These results are also depicted in Figs **3** and **S1**. The proximity of the transposon insertion to the gene start site and the physical location of the mutant in the condensed, ordered library are reported in columns one and two, respectively. CPS biosynthesis was quantified by measuring uronic acid content (μg/ml) and normalized to OD_600_. Mucoviscosity was quantified by sedimentation of bacteria cultured in microplates overnight at 2,000 x *g* for 20 min, where percent hmv is the supernatant OD_600_/total OD_600_ x 100%. Each assay was performed with three or more replicates. Statistically significant differences between WT and each mutant were determined using the Holm-Sidak method, with alpha = 0.05. Computations assumed that all rows were sampled from populations with the same scatter where, * P < 0.05; ** P < 0.01; *** P < 0.001; # P < 0.0001. Note that alternate gene names are reported in parentheses and the transposon insertion in *sdhC* also disrupts the 5’ end of *sdhD*. Genes selected for targeted deletion are identified with Δ.

To validate the utility of the transposon library by identifying both known and novel genes that impact *K*. *pneumoniae* hmv, the condensed library was screened for transposon mutants with reduced hmv using sedimentation assays and the string test (**Fig 1B**). With a hit rate of 2.76%, the 103 mutants initially identified to have reduced hmv based on sedimentation were patched onto LB plates where loss of hmv was confirmed in a secondary screen by string test then a sedimentation assay performed in triplicate (**Fig 1B**). 53 of the 103 primary hits were evaluated in a final sedimentation assay scaled up to a standard culture volume of 3 mL (**S1 Fig**). Ultimately, 44 hypo-mucoviscous (hmv^low^) transposon mutants passed the 3 rounds of screening and confirmation (**Fig 2**). Most of the hits did not stretch at all by string test and were therefore sub-categorized as non-mucoviscous (hmv^0^, 33 hits), leaving 11 hmv^low^ hits that stretched less than five mm by string test (**Figs 2** and **S1**).

**Fig 3:**
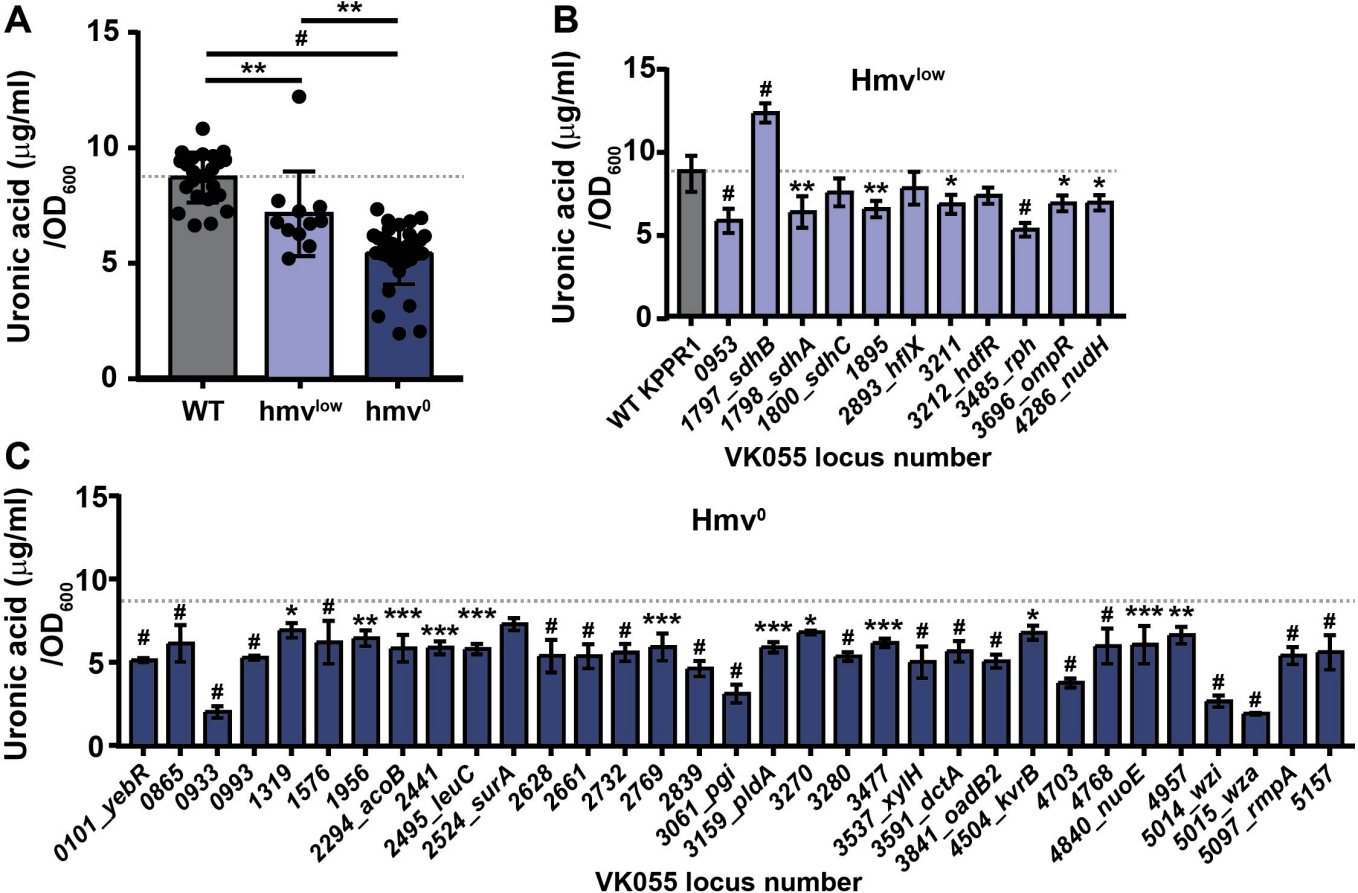
A forward screen identifies transposon mutants that influence *K*. *pneumoniae* hypermucoviscosity (hmv) and capsular polysaccharide production. (A) The mean capsular polysaccharide (CPS) production of 11 hypo-mucoviscous (hmv^low^) and 33 non-mucoviscous (hmv^0^) transposon mutants were quantified in triplicate by measuring uronic acid content and normalized to the optical density at 600 nm (OD_600_). Each marker for wildtype (WT) is an individual replicate (N = 24). Error bars represent one standard deviation from the mean and statistical differences between WT and classes of mutants were determined by unpaired t-test. The mean uronic acid content of each (B) hmv^low^ and (C) hmv^0^ transposon mutant represented by a dot in (A) is shown individually and labeled with the old locus tag number and gene name. Error bars represent one standard deviation from the mean of the assay performed in triplicate. Statistical significance between WT (gray bar or line) and each mutant was determined by unpaired t-test and Holm-Sidak’s multiple comparisons test, with alpha = 0.05. Computations assumed that all rows were sampled from populations with the same scatter where, * P < 0.05; ** P < 0.01; *** P < 0.001; # P < 0.0001. The mean WT value is denoted by a horizontal, dotted gray line.

To investigate the relationship between hmv and CPS production, all 44 transposon mutants were evaluated for capsule (CPS) production by quantifying the amount of uronic acid produced by each strain (**Fig 3**) [32,33]. Overall, both classes of transposon mutants produced significantly less uronic acid than WT, although hmv^0^ hits produced significantly less CPS than hmv^low^ hits (**Fig 3A**). Specifically, 97.0% of the hmv^0^ (N = 32 of 33) and 63.6% of the hmv^low^ (N = 7 of 11) hits synthesized significantly less CPS than WT (**Fig 3**). Notably, hmv^0^ hits encompass a wide range of CPS levels, yet are all non-mucoviscous, and many hmv^low^ strains produce quantities of CPS comparable to hmv^0^ strains, yet retain some mucoviscosity (**Figs 3** and **S1**). Altogether, these data support that CPS production is necessary for *K*. *pneumoniae* to exhibit hypermucoviscosity yet emphasize that other bacterial factors are also likely required for hmv (**Fig 3A**).

The forward transposon screen identified six genes previously identified to support hmv and CPS production, including five hmv^0^ hits (*wzi*, *wza*, *rmpA*, *kvrB*, and *pgi*) and one hmv^low^ hit (*ompR*) (**Fig 3C**) [23,24,34]. These findings serve as internal experimental validation, which provide confidence in our results obtained from screening the transposon library. In addition, the unbiased forward screen identified other genes involved in central metabolism and bacterial cell biology that have not been previously ascribed to impact CPS biosynthesis and hmv. The two classes of genes with the most hits included those involved in cellular metabolism (N = 13) and transport (N = 10), half of which are predicted to have cognate sugar substrates (**Figs 2, 3B and 3C**). The ten transporters identified include five sugar transporters (*VK055_0865*, *VK055_2628*, *VK055_3270*, *xylH*, *VK055_1895*), a C4-dicarboxylate transporter (*dctA*), two amino acid transporters (*VK055_1956* and *VK055_2661*), a nucleoside transporter (*VK055_4768*), and an osmoprotectant transporter (*VK055_4957*) [35,36]. Furthermore, 13 genes that participate in central metabolism were hit including those involved in TCA cycle (*VK055_3477*, *oadB2*, *sdhB*, *sdhA*, *sdhC*), electron transport (*nuoE*), fermentation (*acoB*, *VK055_2769*, *VK055_3280)*, C4-dicarboxylate metabolism (*VK055_0953*), and sugar metabolism (*VK055_0993*, *VK055_2839*, *pgi*), along with many of the aforementioned genes involved in the transport of substrates for these metabolic processes [35,36]. Intriguingly, eight genes related to nucleic acid function were identified including DNA replication, transcription, and RNA biology (*VK055_1319*, *VK055_1576*, *hflX*, *VK055_4703*, *VK055_3211*, *hdfR*, *rph*, and *nudH*), as well as four genes related to protein biology (*yebR*, *VK055_2441*, *leuC*, and *surA*) and one related to lipid biology (*pldA*) [35,36]. Three hypothetical genes were identified, including *VK055_0933*, *VK055_2732*, and *VK055_5157*. Note that the apparent increase in CPS production in the *sdhB* transposon mutant may be confounded by an overt growth defect which dramatically skewed the normalization of uronic acid production to OD_600_; the strain was hypo-mucoviscous when evaluated by string test and sedimentation (**Figs 3B** and **S1A**).

### A reverse phenotypic screen mapped genes linked to capsular polysaccharide biosynthesis and hypermucoviscosity

**Fig 4:**
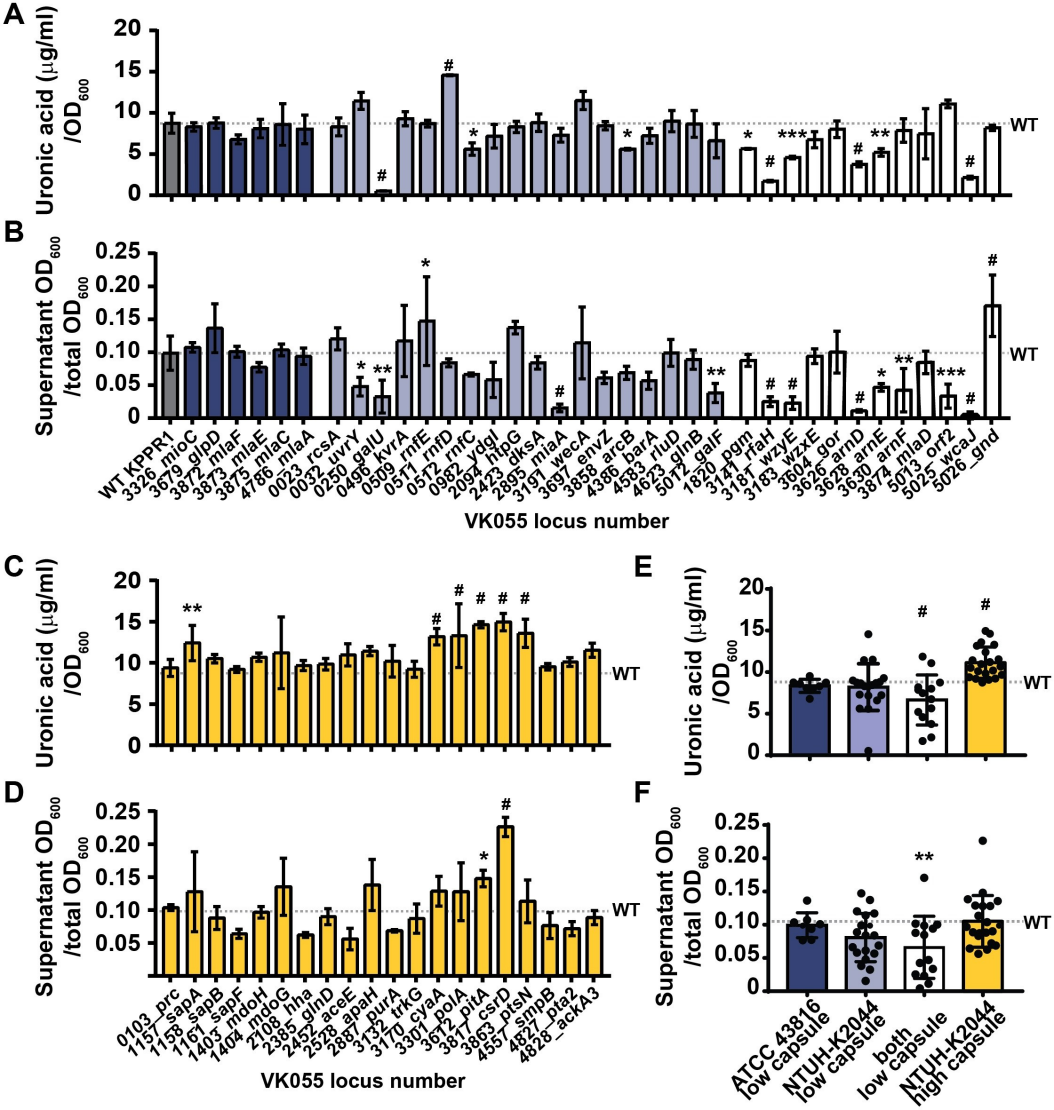
A reverse screen identifies mutants with altered mucoviscosity and capsule levels in KPPR1. Strains reported to have reduced or increased buoyancy were revived from the ordered KPPR1 transposon library. (A, C) The amount of capsule produced by each mutant was quantified by measuring uronic acid content and normalized to the OD_600_. (B, D) The fraction of hypermucoviscous cells that remain in suspension were quantified after low-speed centrifugation of 1 mL overnight culture centrifuged at 7,000 x *g* for 10 min. The x-axis labels in (B, D, F) apply to (A, C, E). The transposon insertion site for each mutant is labeled with the old locus tag number and gene name. Genes that were originally identified to decrease buoyancy in ATCC 43816 are navy, in NTUH-K2044 are light blue, or both are white; genes identified to increase buoyancy in NTUH-K2044 are yellow. All error bars represent one standard deviation from the mean and each assay was performed with three or more replicates. Statistical significance between wildtype (WT) (gray bar or line) and each mutant was determined by unpaired t-test and Holm-Sidak’s multiple comparisons test, with alpha = 0.05 and * P < 0.05; ** P < 0.01; *** P < 0.001; # P < 0.0001. The mean WT value is denoted by a horizontal, dotted gray line. The (E) capsule production and (F) hypermucovisity (7,000 x *g* for 10 min) of each revived KPPR1 transposon mutant was compiled for each reported class of mutants with each circle representing an individual transposon mutant. Error bars represent one standard deviation from the mean and statistical differences between WT [in (E) N = 45 and in (F) N = 30] and classes of mutants were determined by unpaired t-test where, ** P < 0.01 and # P < 0.0001.

A recent study used density-TraDISort to identify *K*. *pneumoniae* transposon mutants with altered buoyancy as a surrogate for CPS production and hmv [23]. This work identified transposon mutants that increased NTUH-K2044 buoyancy, and mutants that decrease NTUH-K2044 and/or ATCC 43816 buoyancy [23]. We sought to integrate the results of the forward screen (**Figs 1, 2** and **3**) with these results by systematically exploring the hmv and CPS production of KPPR1 transposon mutants in genes identified by density-TraDISort. Nine of these genes were primary hits in the forward genetic screen and six passed the secondary and tertiary screens, including *pgi*, *ompR*, *kvrB (mprA)*, *wzi*, *wza*, and *rmpA* (**Figs 1, 2** and **4**). Altogether, 56 mutants identified by density-TraDISort to impact *K*. *pneumoniae* buoyancy [23] were revived from the KPPR1 condensed library and evaluated for hmv by sedimentation and CPS production by uronic acid quantification (**Fig 4**). Twenty of these mutants had been identified to increase buoyancy in NTUH-K2044 and 36 of these mutants had been identified to decrease buoyancy in NTUH-K2044 and/or ATCC 43816 [23]. Note that KPPR1 is a rifampin-resistant derivative of ATCC 43816 [29].

**Fig 5:**
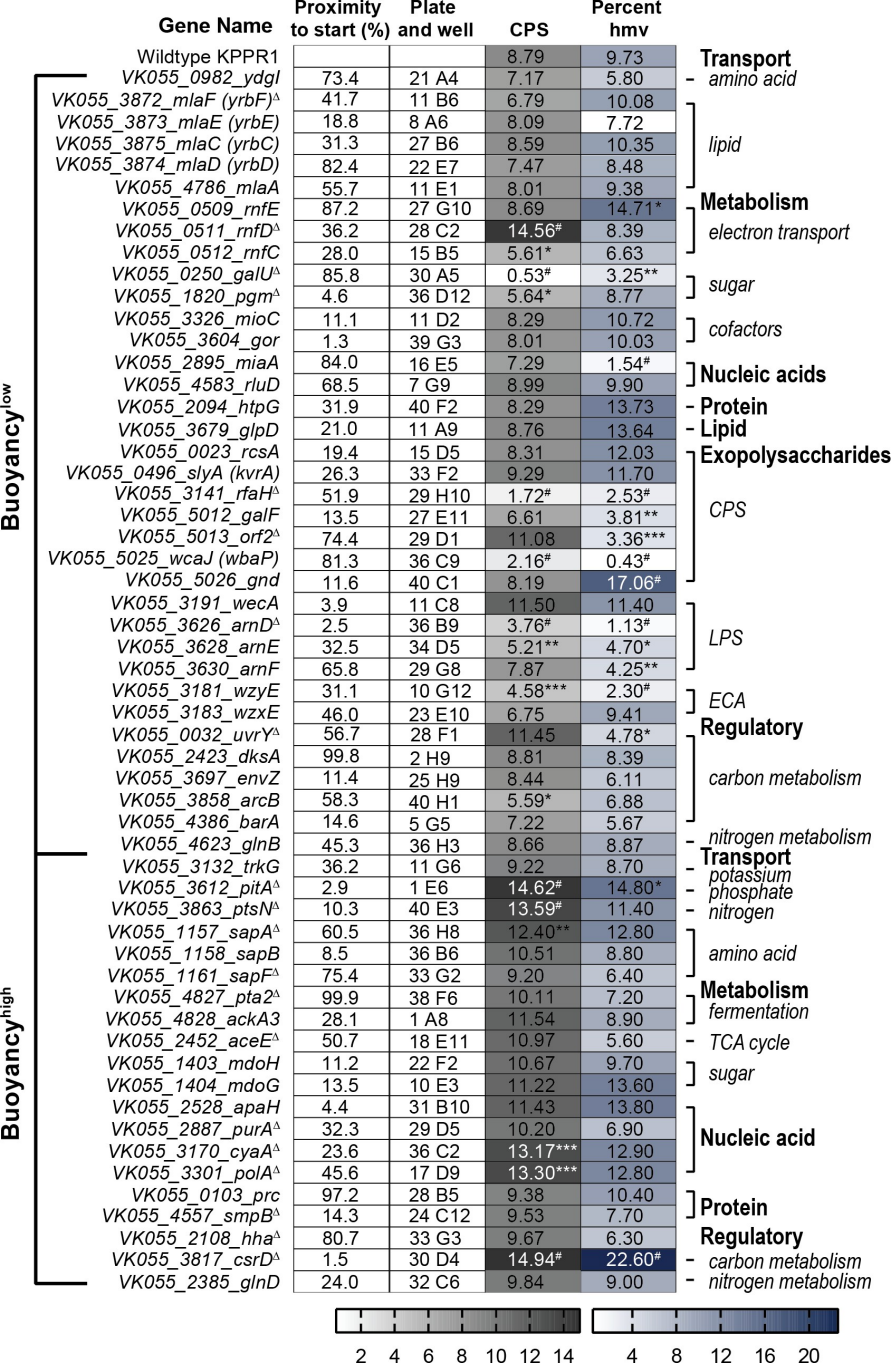
Reverse phenotypic screen of KPPR1 hypermucoviscosity and capsular polysaccharide biosynthesis. Shown are the results of the reverse screen categorizing genes that influence hypermucoviscosity (hmv) and/or capsular polysaccharide (CPS) biosynthesis, where the predicted gene function is annotated in the right margin. These results are also depicted in Fig 4. The proximity of the transposon insertion to the gene start site and the physical location of the mutant in the condensed, ordered library are reported in columns one and two, respectively. CPS biosynthesis was quantified by measuring uronic acid content (μg/ml) and normalized to OD_600_. Mucoviscosity was quantified by sedimentation of 1 ml of bacterial culture at 7,000 x *g* for 10 min, where percent hmv is the supernatant OD_600_/total OD_600_ x 100%. Each assay was performed with three or more replicates. Statistically significant differences between WT and each mutant were determined using the Holm-Sidak method, with alpha = 0.05. Computations assumed that all rows were sampled from populations with the same scatter where, * P < 0.05; ** P < 0.01; *** P < 0.001; # P < 0.0001. Note that KPPR1 is a rifampin resistant derivative of ATCC 43816 and alternate gene names are reported in parentheses. Genes selected for targeted deletion are identified with Δ.

Of the 36 genes predicted to decrease CPS production, 14 had significantly reduced hmv and/or CPS. Six were significantly hmv^low^/CPS^low^ (*galU*, *rfaH*, *wzyE*, *arnD*, *arnE*, and *wcaJ*), three were hmv^WT^/CPS^low^ (*rnfC2*, *arcB*, and *pgm*), and five were hmv^low^/CPS^WT^ (*uvrY*, *miaA*, *galF*, *arnF*, and *orf2*); surprisingly, one was hmv^WT^/CPS^high^ (*rnfD*) and two were hmv^high^/CPS^WT^ (*rnfE* and *gnd*) (**Figs 4A, 4B** and **5**). Of the 20 genes previously identified to increase CPS production in NTUH-K2044, two were hmv^high^/CPS^high^ in KPPR1 (*pitA* and *csrD*), while four were hmv^WT^/CPS^high^ (*sapA*, *cyaA*, *polA*, and *ptsN*) (**Figs 4C, 4D** and **5**). Intriguingly, ten transposon mutants trended toward hmv^low^/CPS^high^ (*uvrY*, *rnfD*, *orf2*, *sapB*, *hha*, *aceE*, *purA*, *smpB*, *pta2*, and *ackA3*); although, for most of these strains, CPS production and/or hmv were not significantly different from WT (**Fig 4A–4D**). Overall, transposon insertions in genes previously determined to reduce buoyancy in both ATCC 43816 and NTUH-K2044, were most likely to reduce hmv or CPS in KPPR1 (8 of 12, 66.7% validation) (**Figs 4A, 4B** (white bars)**, 4E, 4F** and **5)**. This means that only six other genes (out of 24, 25.0%) previously identified to reduce buoyancy in ATCC 43816 or NTUH-K2044 validated with the KPPR1 transposon mutants present in the condensed library [(**Figs 4A, 4B,** (navy and light blue bars) and **5**]. These results emphasize that genes identified across multiple strains are more likely to be integral to CPS biosynthesis and hmv biology species-wide. Furthermore, when evaluated as a whole group, genes previously identified to increase buoyancy significantly increased CPS levels, but not hmv, in KPPR1 transposon mutants (**Fig 4E and 4F**). In total, these results echo what was observed in the forward screen, that hmv and CPS overproduction are two distinguishable phenotypes (**Figs 3** and **4**).

### Hypermucoviscosity and CPS production are coordinated, but dissociable

All together, the forward and reverse screens quantified both hmv and CPS production in 100 transposon mutants and identified 45 hmv^low^/CPS^low^ mutants, three hmv^WT^/CPS^low^ mutants, nine hmv^low^/CPS^WT^ mutants, two hmv^high^/CPS^WT^ mutants, one hmv^WT^/CPS^high^ mutant, one hmv^low^/CPS^high^ mutant, and two hmv^high^/CPS^high^ mutants. These data provide a rich resource for examining if *K*. *pneumoniae* CPS production and hmv are indeed interconnected. The nonparametric Spearman correlation coefficient between uronic acid concentration and sedimentation efficiency for all 100 mutants examined was *r*^2^ = 0.5924 (p < 0.0001), identifying a significant link between the two variables.

**Fig 6:**
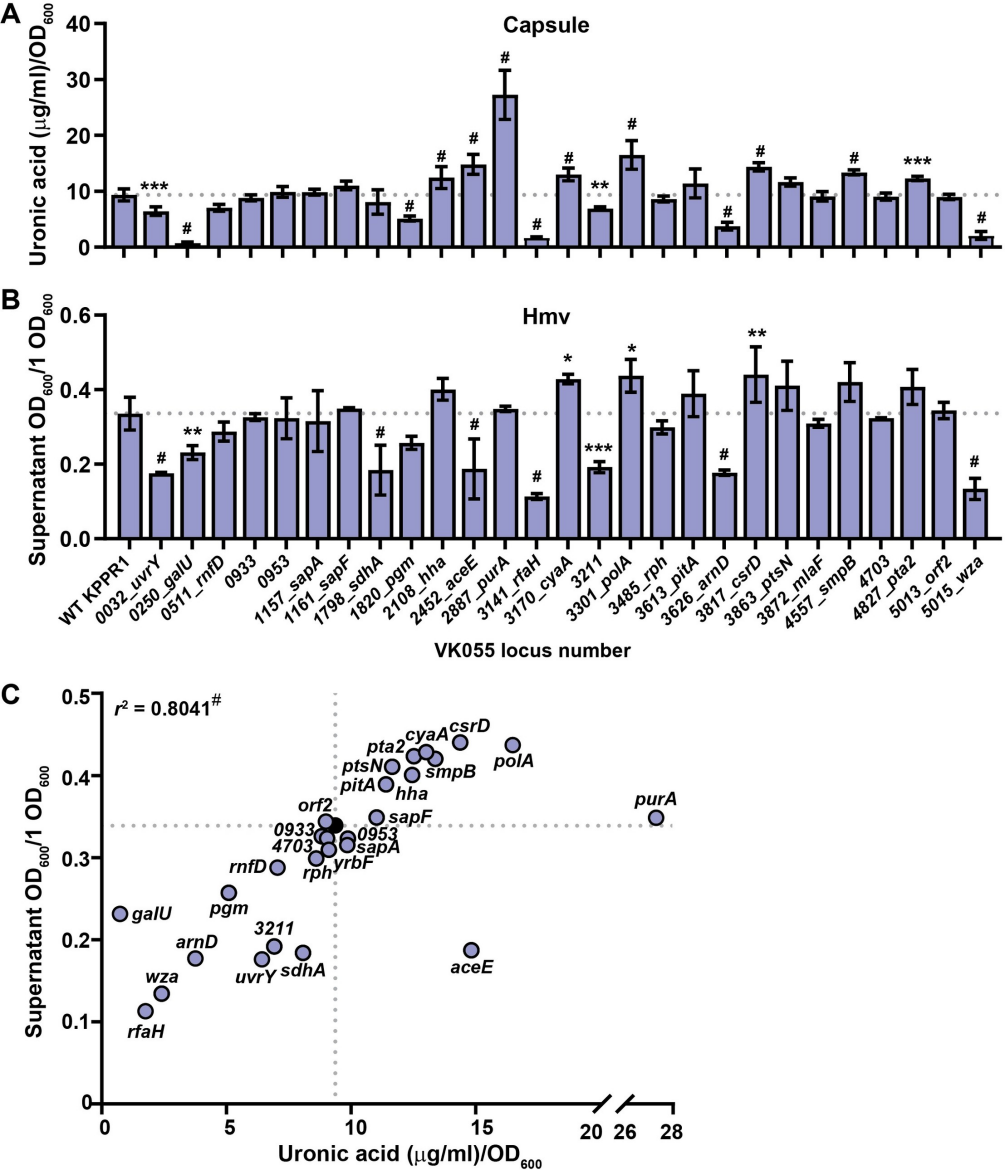
Hypermucoviscosity and capsular polysaccharide levels are coordinated, but dissociable. (A) The amount of capsule produced by 27 targeted deletion mutants was quantified by measuring uronic acid content and normalized to the OD_600_. (B) The fraction of hypermucoviscous cells remaining in suspension were quantified after low-speed centrifugation (1,000 x *g* for 5 min) of 1 OD_600_ unit of cells resuspended in 1 ml of PBS. All error bars represent one standard deviation from the mean and each assay was performed with six or more replicates. Statistical significance between wildtype (WT) and each mutant was determined by unpaired t-test and Holm-Sidak’s multiple comparisons test, with alpha = 0.05. (C) Data from A and B were coordinately plotted on a single graph and labeled with the gene name or gene number (VK055_XXXX). The nonparametric Spearman correlation coefficient for all targeted deletion mutants is r^2^ = 0.8041^#^. All computations assumed that data were sampled from populations with the same scatter where, * P < 0.05; ** P < 0.01; *** P < 0.001; # P < 0.0001. The mean WT value is denoted by dotted gray lines and a black marker.

To confirm that the phenotypes observed in the transposon mutants identified in the forward and reverse screens are attributable to the disrupted gene, a subset of these 100 transposon mutants were identified for further study. Twenty-seven representative transposon insertions were selected for targeted gene deletion based on having diverse combinations of CPS production and hmv (**Figs 2, 3, 4** and **5**). The resulting isogenic mutants were then systematically evaluated for CPS production and hmv (**Fig 6A and 6B**) [30]. Seventeen isogenic mutants (63.0%) exhibited significantly altered CPS production and hmv similar to the corresponding transposon mutant. These 17 isogenic mutants fell into six categories: (1) hmv^low^/CPS^low^ (Δ*uvrY*, Δ*galU*, Δ*rfaH*, Δ*VK055_3211*, Δ*arnD*, Δ*wza*), (2) hmv^WT^/CPS^low^ (Δ*pgm*), (3) hmv^WT^/CPS^high^ (Δ*hha*, Δ*aceE*, Δ*purA*, Δ*smpB*, Δ*pta2*), (4) hmv^low^/CPS^WT^ (Δ*sdhA*), (5) hmv^high^/CPS^high^ (Δ*cyaA*, Δ*polA*, Δ*csrD*), and (6) hmv^low^/CPS^high^ (Δ*aceE*) (**Fig 6**). The hmv and CPS quantification data from Fig 6A and 6B were aggregated on a single X-Y plot to evaluate the relationship between hmv and CPS production in the targeted deletion mutants (**Fig 6C**). The nonparametric Spearman correlation coefficient between uronic acid concentration and sedimentation efficiency for all 27 targeted deletion mutants was *r*^2^ = 0.8041 (p < 0.0001), again supporting the historical perspective that CPS production and hmv are interconnected processes. However, it is notable that several mutants only had one parameter significantly change (Δ*sdhA*, Δ*purA*, Δ*pgm*, Δ*hha*, and Δ*smpB*) or, surprisingly, had CPS production and hmv significantly altered in opposite directions (Δ*aceE*) (**Fig 6**). Moreover, we did not identify any targeted deletion mutants with increased hmv and reduced or WT levels of CPS biosynthesis, supporting the requirement of CPS biosynthesis for hmv.

Six mutants (Δ*galU*, Δ*wza*, Δ*purA*, Δ*csrD*, Δ*sdhA*, Δ*aceE*), representing an array of altered CPS and hmv levels, were complemented *in trans* with the deleted gene under the control of its native promoter (**S2A–S2D Fig)**. The Δ*aceE* mutant required a slightly altered complementation vector backbone as the strain is exquisitely sensitive to chloramphenicol. Instead of using the pACYC184Δ*tet* backbone, Δ*aceE* was complemented with the pACYC184Δ*cat* backbone (**S2C and S2D Fig**). For five mutants, the complementation vector restored hmv or CPS production to WT levels, indicating that the phenotypes of these strains are not due to secondary mutations in the chromosome, which may occur with lambda Red recombinase (**S2A–S2D Fig**).

Although pACYC184Δ*tet* is a low copy number plasmid, complementing Δ*sdhA in trans* significantly reduced CPS levels and hmv compared to WT or Δ*sdhA* with vector alone (**S2A and S2B Fig**). Analysis of the succinate dehydrogenase locus with Softberry BPROM and FgenesB predicts that s*dhA* is the third gene in a four gene operon with two predicted promoters [37]. Despite using various combinations of genes and promoters, we have been unable to generate a complementation vector that restores Δ*sdhA* hmv to WT levels. It is possible that the regulation of the locus is complex and intracellular ratios of each gene in the operon finely tune the effect of succinate dehydrogenase function on hmv and CPS biosynthesis. To evaluate if the loss of hmv in Δ*sdhA* is likely due to the targeted deletion of *sdhA* or off-target effects due to lambda Red recombinase activity, we generated four additional isogenic Δ*sdhA* mutants (isolates #2, 7, 9, and 11). All four isolates exhibited the same hmv and CPS production as the original Δ*sdhA* mutant (isolate #1) (**S2E and S2F Fig**).

### Capsular polysaccharide chain length correlates with uronic acid content, but not hypermucoviscosity in KPPR1

**Fig 7:**
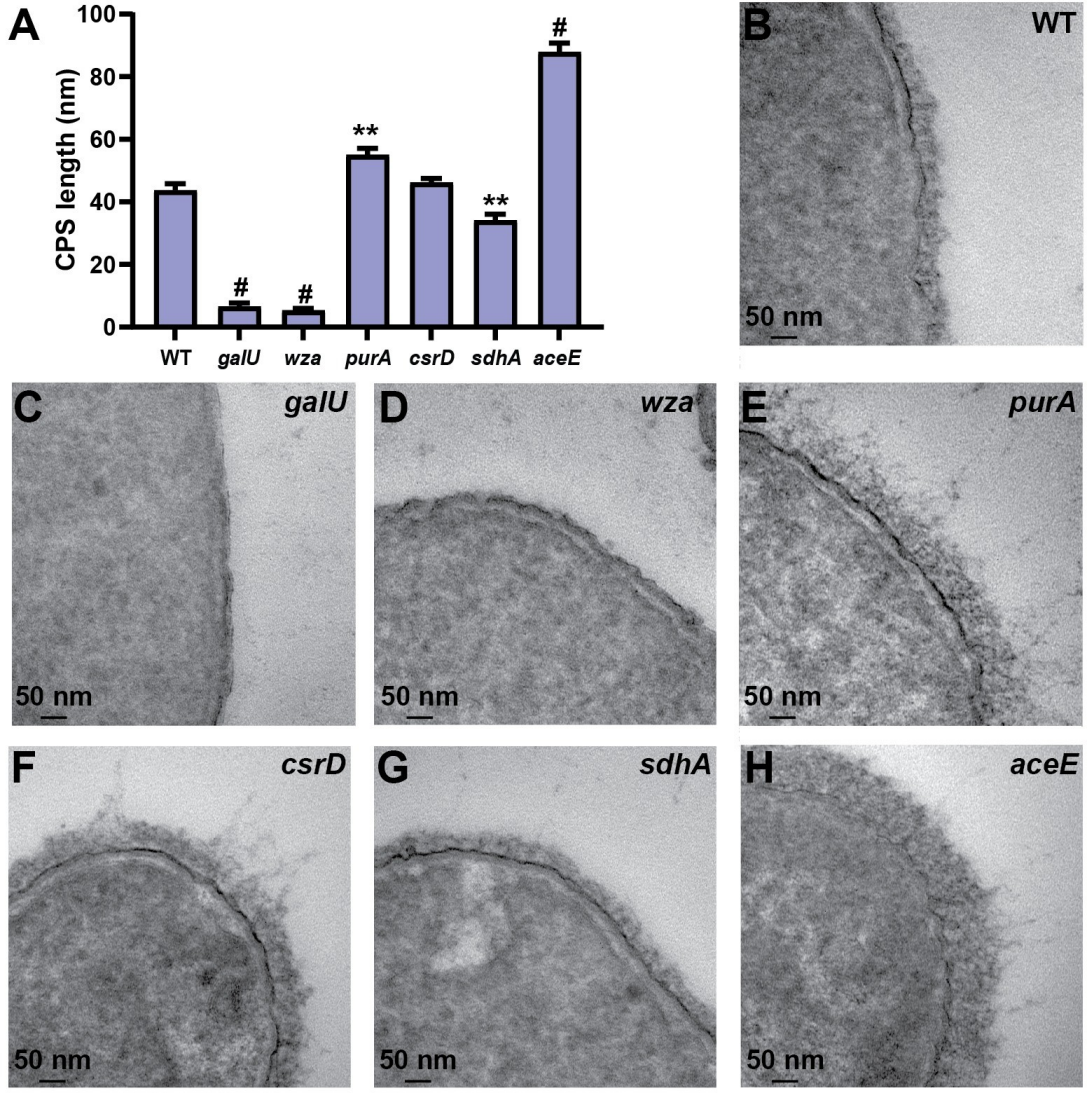
Electron micrographs of select mutants reveal that capsular polysaccharide length reflects uronic acid content, not hypermucoviscosity. Wildtype (WT) and six representative mutants were imaged using transmission electron microscopy. (A) Capsular polysaccharide (CPS) length was measured in FIJI ImageJ 1.53c. (B-H) One representative image for each strain is shown. Data shown in Fig 6 classify Δ*galU* and Δ*wza* hmv^low^/CPS^low^, Δ*purA* as hmv^WT^/CPS^high^, Δ*csrD* as hmv^high^/CPS^high^, Δ*sdhA* as hmv^low^/CPS^WT^, and Δ*aceE* as hmv^low^/CPS^high^. Error bars represent the standard error of the mean from 24 individual measurements collected from 2–6 images at 40,000x magnification. Statistical differences between WT and each mutant was determined by unpaired t-test and Holm-Sidak’s multiple comparisons test, where ** P < 0.01 and # P < 0.0001.

It was recently shown that specific point mutations in *wzc* increase CPS polymerization and mucoviscosity in carbapenem-resistant isolates of *K*. *pneumoniae*, which lack the *rmp* locus [38]. These point mutations enhanced resistance to macrophage phagocytosis, increased lethality in a blood stream infection model of zebrafish larvae, and increased dissemination from the urinary tract in a murine model of UTI [38]. However, other studies have shown that the *rmp* locus encodes two proteins, RmpC and RmpD, which independently increase CPS biosynthesis and hmv, respectively [26,27]. To discriminate whether CPS polymerization or other cellular factors were responsible for altered hmv in the targeted deletion strains generated here, six mutants encompassing a variety of hmv and CPS combinations, including hmv^low^/CPS^low^ (Δ*galU* and Δ*wza*), hmv^WT^/CPS^high^ (Δ*purA*), hmv^high^/CPS^high^ (Δ*csrD*), hmv^low^/CPS^WT^ (Δ*sdhA*), and hmv^low^/CPS^high^ (Δ*aceE*) were imaged using transmission electron microscopy (**Fig 7**). The thickness of the CPS was quantified using ImageJ (**Fig 7A)**. The measured CPS thickness corresponded to the uronic acid content reported in **Fig 6A,** but not the sedimentation efficiency (**Fig 6B**). These data support recent evidence that in *rmp* encoding strains, hmv requires some facet of capsule biosynthesis, yet is a distinct cellular process [27].

### Distinct function of hypermucoviscosity and capsular polysaccharide in cell association and serum survival

**Fig 8:**
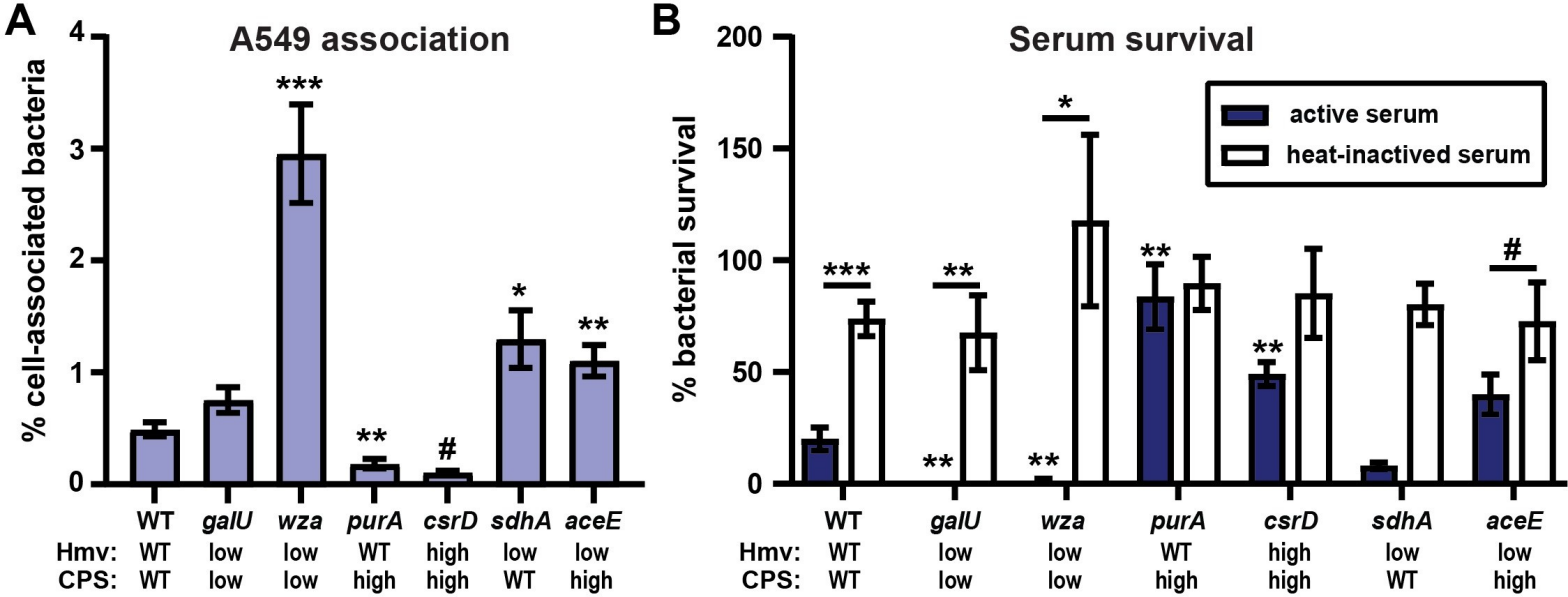
Hypermucoviscosity blocks cell association while capsular polysaccharide blocks serum-mediated killing. The ability of WT and six representative mutants to (A) associate with A549 human lung epithelial cells and (B) resist serum killing was evaluated. All error bars represent the standard error of the mean from at least three independent experiments. Statistical significance between wildtype (WT) and each mutant was determined by unpaired t-test and Holm-Sidak’s multiple comparisons test, where * P < 0.05; ** P < 0.01; *** P < 0.001; # P < 0.0001. In (B) statistical significance between active and heat-inactivated serum was also calculated by the same method.

It is well-established CPS protects bacteria from complement-mediated killing in the serum [39–44]. In addition, acapsular *K*. *pneumoniae* strains have been shown to associate more with PMNs, macrophages, and some epithelial cell lines [38,39,45,46]. However, recent data have shown that WT KPPR1S and *rmpC* (hmv^WT^/CPS^low^) exhibit similar adherence and internalization to bone marrow-derived macrophages, while *rmpD* (hmv^low^/CPS^WT^) exhibits increased adherence and internalization to the macrophage-like J774A.1 cell line, compared to WT KPPR1S [17,26]. These data suggest that hmv blocks adherence and internalization by macrophages. To further explore whether CPS and hmv have distinct roles in host-pathogen interactions, the ability of six representative mutants of varying hmv and CPS combinations were examined for their ability to associate with immortalized lung epithelial cells (A549) and survive serum exposure (**Fig 8**). In general, strains with reduced hmv exhibited significantly increased association with A549 cells (**Fig 8A**). Most notably, Δ*sdhA* and Δ*aceE*, which make CPS, but have reduced hmv, associated more with A549 cells, while Δ*csrD*, which is hmv and makes a similar amount of CPS to Δ*aceE*, had reduced cell association (**Fig 8A**). These data recapitulate the increased association of the encapsulated, but non-mucoviscous *rmpD* mutant with J774A.1 cells. Conversely, mutant strains that produce less CPS than WT were more sensitive to serum killing than strains that produce CPS levels greater than or comparable to WT (**Fig 8B**). In particular, Δ*sdhA* and Δ*aceE*, both of which are less mucoviscous than WT, survived serum comparably to WT (**Fig 8B**). Altogether, these data strengthen the emerging model that hmv blocks bacterial association with host cells, while CPS protects bacteria in the serum.

### Isogenic mutants with altered CPS production and hypermucoviscosity are less fit in a murine pneumonia model

**Fig 9:**
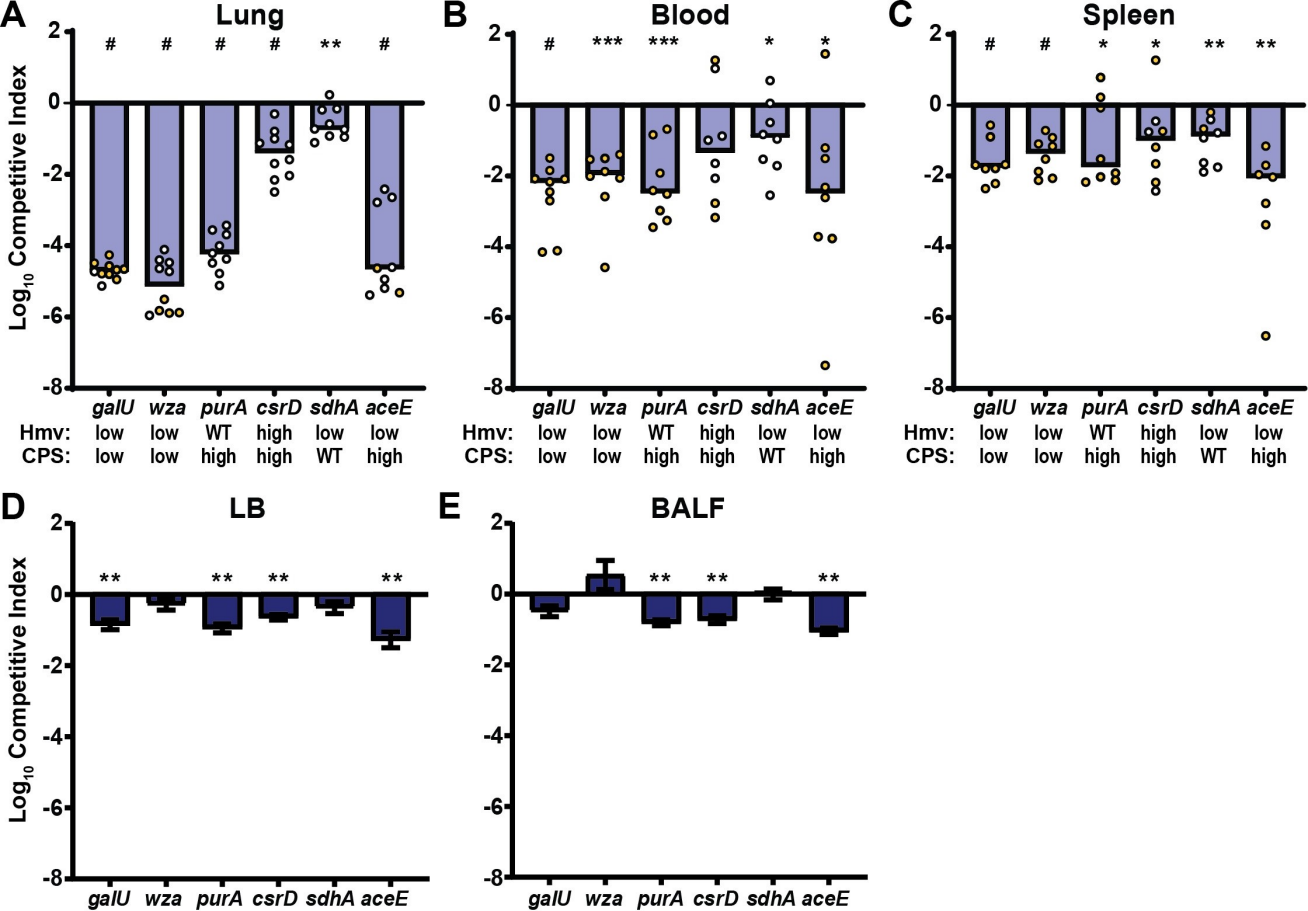
The competitive fitness of select mutants is more attenuated in a murine model of disseminating pneumonia than *in vitro*. (A-C) C57BL/6 mice were infected retropharyngeally with 1 x 10^6^ colony forming units (CFU) of a 1:1 ratio of wildtype (WT) to mutant, where each mutant and its significantly different levels of hypermucoviscosity (hmv) and capsular polysaccharide (CPS) are identified on the x-axis. The input ratios were determined by differential plating. After 24 h of infection, the bacterial burdens of WT and each mutant in the (A) lungs, (B) blood, and (C) spleens were determined by differential plating. Each dot represents an individual mouse and yellow dots indicate that no mutant was detected in the outputs. The competitive fitness of each mutant against WT was also evaluated *in vitro* after culturing in (D) LB and (E) BALF for 24 h. The limit of detection was 100 CFU/ml. Competitive indices were calculated by dividing the output ratio of mutant/WT by the input ratio of mutant/WT. All competitive indices were log_10_-transformed and any significant differences from a competitive index of 0 was determined by a one-sample t test where, * P < 0.05; ** P < 0.01; *** P < 0.001; # P < 0.0001.

It is well-established that *K*. *pneumoniae* requires CPS to be fully virulent in multiple models of infection, including pneumonia and UTI [21,22]. We hypothesized that both CPS production and hmv are important for full virulence and that disconnecting the two processes may reduce *in vivo* fitness. To test this hypothesis, the same six mutant strains examined above (Δ*galU*, Δ*wza*, Δ*purA*, Δ*csrD*, Δ*sdhA* and Δ*aceE*) were competed against WT KPPR1 in a murine model of disseminating pneumonia [30]. Mice were inoculated retropharyngeally with a targeted input ratio of 1:1 WT:mutant. At 24 h post-infection, bacterial burdens of WT and mutant in the lungs, blood and spleens were enumerated and the competitive indices were calculated (**Figs 9** and **S3**). All mutants were significantly out-competed *in vivo*. Δ*galU* (-4.71 log), Δ*wza* (-5.14 log), Δ*purA* (-4.18 log), and Δ*aceE* (-4.21 log) were all dramatically out-competed in the lung (**Fig 9A**) and did not disseminate into the blood and spleens, as mutant CFUs were below the limit of detection in these organs (**Figs 9B, 9C** and **S3**). Δ*csrD* (-1.44 log) and Δ*sdhA* (-0.57 log) had less dramatic decreases in competitive fitness in the lungs (**Fig 9A**) and were still able to disseminate into the blood and spleens of several mice (**Figs 9B, 9C** and **S3**).

Although all mutants were significantly outcompeted *in vivo*, they exhibited diverse growth phenotypes in LB medium (**S4 Fig**). *In vitro*, only Δ*aceE* had a significantly longer doubling time than WT (3.46-fold) (**S4A and S4B Fig**). In addition, all strains except Δ*csrD* yielded less total bacterial growth than WT *in vitro*, as quantified by the area under the growth curve; although significant, these differences were quite subtle (**S4C Fig**). These growth defects make it difficult to ascertain if *in vivo* fitness defects are due to an *in vivo* growth defect. To parse whether the reduced relative output of mutant strains from the *in vivo* murine competitions is primarily due to a competitive growth defect or an *in vivo* fitness defect, the growth of each mutant strain in competition with WT was quantified *in vitro* in LB medium and bronchoalveolar lavage fluid (BALF) (**Fig 9D and 9E**). While many mutants were significantly out-competed *in vitro*, all were out-competed 0.5–5 logs greater *in vivo*, suggesting that some host-specific factors (e.g. immunological or nutrient-availability) also influence the competitive fitness of the mutants *in vivo*. Altogether, these results suggest that appropriate coordination of CPS biosynthesis, hmv, and central metabolism is required for optimal growth and fitness in a murine pneumonia model.

## Discussion

CR-cKp tops the list of urgent antibiotic-resistant threats most recently released by the CDC [47]. Moreover, the growing incidence of hvKp and the emergence of CR-hvKp emphasizes the looming threat that *K*. *pneumoniae* poses to human health [3]. Historically, the hypervirulence of hvKp has been primarily attributed to RmpA-mediated increased production of CPS, along with stealth siderophores. These strains can often be identified by a positive string test, demonstrating hypermucoviscosity [3,5]. The hmv of hvKp is generally ascribed to increased production of CPS; however, recent studies have challenged this model [24,26,27,34,48]. A shift in our understanding of *K*. *pneumoniae* hmv has emerged in recent years as genes, namely *rmpC* and *rmpD*, have been identified that differentially affect hmv and CPS biosynthesis [26,27]. Here, we have generated a condensed, ordered transposon library in a genetically tractable and hmv strain of *K*. *pneumoniae*, KPPR1, and used this library to investigate a question at the forefront of *K*. *pneumoniae* pathogenesis, namely, how do hmv and CPS biosynthesis independently and coordinately impact invasive hvKp infections?

In total, we quantified the impact of 100 transposon insertion mutants and 27 targeted gene deletions on both CPS production and hmv. The relationship between these two properties was examined and the nonparametric Spearman correlation coefficient for CPS production and hmv was significant for both the transposon mutants (*r*^2^ = 0.5924; P < 0.0001) and targeted gene deletion strains (*r*^2^ = 0.8041; P < 0.0001). These data support the long-held view that hmv and CPS production are inter-related. However, several transposon and targeted deletion mutants dissociate CPS production and hmv, also supporting the emerging perspective that CPS is not the only biochemical feature driving hmv [17]. Furthermore, we collected TEM images of 3 targeted gene deletion mutants (Δ*aceE*, Δ*sdhA*, and Δ*purA*) with disproportionate CPS production and hmv, along with Δ*galU* and Δ*wza* (hmv^low^/CPS^low^) and Δ*csrD* (hmv^high^/CPS^high^) and found that CPS thickness correlated with the uronic acid content produced by each strain, not the hypermucoviscosity (**Figs 6** and **7**). In addition, some transposon mutants exhibited phenotypes that trended toward increasing CPS production while reducing hmv (**Figs 2, 3, 4** and **5**). Considering these data altogether, we propose that in order to exhibit hmv, *K*. *pneumoniae* requires CPS along with other biochemical factors.

Several genes related to the TCA cycle, pyruvate metabolism, and cellular energetics appear to decouple CPS biosynthesis and hmv. This suggests that hmv and CPS biosynthesis are integrated with the metabolic status of the cell. Further studies are required to dissect the metabolic pathways that control the biosynthesis of CPS and hypermucoviscosity. Moreover, the ability of the bacteria to distinctly control hmv and CPS biosynthesis suggest that there are environmental conditions in which one or both properties are advantageous, emphasizing that while these two features are closely associated with hypervirulent *K*. *pneumoniae*, they likely serve distinct functions within specific environments and may actually be regulated in response to the local environment [38]. For example, the hmv^low^/CPS^WT^ Δ*rmpD* mutant adheres to macrophage-like J774A.1 cells more than WT, while the hmv^WT^/CPS^low^ Δ*rmpC* mutant adheres similar to WT [26,27]. Here, we have shown that targeted deletion strains with reduced hmv exhibit increased *K*. *pneumoniae* association with human lung epithelial cells (**Fig 7A**). However, targeted deletion strains that increase CPS biosynthesis improved resistance to human serum, while those with reduced CPS production were more sensitive to killing by human serum (**Fig 7B**). These data provide two examples in which host-pathogen interactions may be distinctly impacted by hmv or CPS production. A recent study found that non-mucoviscous strains are more likely to be isolated from urine than blood [38]. It seems quite possible that adherence to uroepithelial cells in an environment subject to high urine flux is critical and that hmv may be a fitness disadvantage for *K*. *pneumoniae* strains in the urinary tract. Conversely, during invasive infections, strains exhibiting increased hmv may have a fitness advantage by avoiding adherence to host macrophages.

To begin understanding the individual roles of hmv and CPS within the context of the host with the mutants identified here, we evaluated the competitive fitness of the six representative targeted gene deletion mutants (Δ*aceE*, Δ*sdhA*, Δ*purA*, Δ*galU*, Δ*wza*, and Δ*csrD*) *in vitro* in LB and BALF, as well as *in vivo* in a murine model of pneumonia. *In vitro*, Δ*csrD*, Δ*aceE*, and Δ*purA* were significantly out-competed by WT in both LB and BALF; although all competitive defects were less than -1.27 log (**Fig 9D and 9E**). However, *in vitro* competitive defects did not necessarily recapitulate *in vivo* fitness defects, as Δ*csrD*, Δ*aceE*, and Δ*purA* were significantly out-competed by WT in the murine lung by -1.44, -4.21, and -4.18 log, respectively. Such data suggest that both nutrient-related and host-specific factors drive the loss of *in vivo* fitness for Δ*aceE* and Δ*purA*, while the *in vivo* fitness defect of Δ*csrD* may be primarily driven by nutrient utilization. Conversely, both Δ*galU* and Δ*wza* competed equally well against WT in BALF yet exhibited major fitness defects in lung colonization and dissemination to the blood and spleens in the murine pneumonia model (**Fig 9**); these data support the established importance of hmv and capsule *in vivo*, especially for invasive infections (**Fig 9**) [38,46,49,50]. Note that the dramatic *in vivo* competitive defect of Δ*wza* may be compounded by the loss of surface exposed CPS and cell envelope stress caused by the accumulation of capsule intermediates in the periplasm (**Fig 9**) [46]. It is important to note that four of the mutants tested, Δ*csrD*, Δ*aceE*, Δ*sdhA*, and Δ*purA*, impact both hmv and/or CPS biosynthesis and are involved in central metabolism, complicating any definite conclusions about how their protein products contribute to *in vivo* fitness. In fact, other purine biosynthesis mutants (*purF*, *purL*, *purH*) have been identified to have a fitness defect in *K*. *pneumoniae*; although, the impact of these other genes on CPS production and hmv was not evaluated [30]. The predicted alterations in carbon metabolism and cellular redox status in these mutants may itself alter the *in vivo* fitness of *K*. *pneumoniae*, but it is also possible that the observed fitness defects are due to altered CPS biosynthesis and hmv. It is intriguing that the two mutants (Δ*csrD* and Δ*sdhA*) that retain the ability to disseminate from the lungs are those that maintain ratios of CPS to hmv most similar to WT (**Figs 6** and **9**). This observation suggests that both CPS production and hmv may be important for invasive *K*. *pneumoniae* infections, which has been observed clinically [38]. Nonetheless, further studies are needed to identify the precise signals that regulate CPS biosynthesis and hmv, as well as dissect how these regulatory pathways overlap, diverge, and impact pathogenesis.

Some novel pathways identified here that should be of immediate focus are the succinate dehydrogenase complex and the hypothetical gene *VK055_3211* and its divergently transcribed regulator, *VK055_3212_hdfR* [51,52]. Homologues to *VK055_3211* and *hdfR* have been identified in *E*. *coli* to contribute to the organization of the Ori region during chromosome replication and HdfR has been shown to repress the flagellar master operon (*flhDC*) [51,53]. Although KPPR1 does not encode *flhDC*, *hdfR* expression is repressed by H-NS, which has been shown to repress hmv and CPS biosynthesis in *K*. *pneumoniae* [53,54]. Altogether, these data suggest that HdfR may be another component of the complex CPS biosynthesis and hmv regulatory networks in *K*. *pneumoniae* and may coordinate these features with cell replication. More globally, the identification of genes linked to central metabolism that, when disrupted, result in a decrease in hmv suggests that hmv is tightly linked to the energy status of the cell. This is not too surprising as elaborating large extracellular macromolecules is an energetically expensive process and has been showed to serve as an energy reservoir in other bacterial species [55–57]. It is intriguing that many of these hits result in complete loss of hmv, while retaining intermediate levels of CPS production (**Fig 3**). The stronger effect of perturbing bioenergetics on hmv than CPS may explain why these genes have not previously been identified to impact CPS biosynthesis.

Further strengthening the connection between the integration of cellular metabolism with the regulation of CPS and hmv, is our confirmation that several genes involved in the carbon storage regulatory network coordinately increase buoyancy, where BarA/UvrY and DksA oppose CsrD and CyaA activity [23,58]. By systematically quantifying hmv and CPS production in transposon mutants previously identified to impact buoyancy in NTUH-K2044 [23], we have confirmed that transposon insertions in *uvrY* significantly decreases hmv, *cyaA* increases CPS, and *csrD* increases both CPS biosynthesis and hmv in KPPR1 (**Fig 4A–4D**). The carbon storage regulatory network interfaces with the cAMP receptor protein, CRP, which has been previously shown to repress CPS biosynthesis at the transcriptional level in NTUH-K2044 and CG43 [59,60]. On the other hand, of all the tested genes in the *sapABCDF* cationic peptide ABC transporter operon, which had been identified to increase buoyancy in NTUH-K2044, only *sapA* significantly increased CPS levels in KPPR1 (**Fig 4A–4D**). Altogether these results suggest that the carbon storage regulatory circuit may represent a more broadly conserved mechanism *K*. *pneumoniae* employ to control hmv and CPS biosynthesis, while the *sap* operon may exert control of these processes in clonal groups more closely related to NTUH-K2044. It is important to appreciate that while the density-TraDISort study was only able to identify transposon mutants that increased CPS production in NTUH-K2044, many had a similar effect in KPPR1. The authors did note that several genes including *uvrY*, *barA*, *csrB*, *rcsA* and *rcsB* met some, but not all of their screening criteria to be identified as hits in ATCC 43816 [23]. Altogether, the results of the forward and reverse screens further support the notion that CPS biosynthesis and hmv are tightly linked to the metabolic state of *K*. *pneumoniae* and that although hmv requires CPS production, it is not the only factor. Therefore, it is critical to continue to evaluate both of these virulence-associated features so that biological effects on each process may be assessed independently. This may be accomplished by focusing on hits identified here that only affect hmv or CPS biosynthesis, or in some cases impose an opposite effect on these two properties. It may be that changes in the intracellular pools of metabolic intermediates or signaling nucleotides in response to environmental oxygen, carbon- or nitrogen-sources differentially regulate hmv and CPS.

The reverse screen executed here built on a recent density-TraDISort study that identified transposon mutants with altered buoyancy in NTUH-K2044 and/or ATCC 43816, the parental strain of KPPR1 [23]. For those transposon mutants that did not reproduce the previously reported phenotype, it is important to appreciate that the two screens are experimentally distinct in that one was performed by separating a pool of mutants over a Percoll gradient and the other probed each mutant individually using the sedimentation assay. Some mutants may conceivably behave differently when assayed in a pool versus individually. This is especially true if the product of the mutated gene can be complemented by other mutants in the pool that are effectively WT for the gene of interest. Alternatively, it is possible that the transposon mutants in the KPPR1 library are not relevant under the experimental conditions or functionally inactivating. However, the site of transposon insertion ranged from 1.3–99.8% from the predicted start codon, with a median value of 34.35% (17.75–66.48% interquartile range). Thus, most transposon mutants are expected to be functionally disrupted (**Fig 5**). For those genes that did not exhibit an effect on CPS or hmv, the median distance from the start site was 31.3% (17.75–45.825% interquartile range), indicating that most negative results skewed toward the start codon. This observation was surprising as we anticipated that transposon insertions toward the end of the gene, would be more likely to have less impact on function. This expectation likely over-simplifies the complexities of protein function and operon structure and suggests that many of the transposon mutants in the condensed library provide valuable biological insights, regardless of their distance from the start site. It may even be valuable to return to the full transposon library to access multiple transposon insertion sites in the same gene or operon, thereby providing a comparison of similar, but unique mutants. Even fuller datasets may be achieved by accessing the two other ordered *K*. *pneumoniae* transposon libraries in addition to the one generated here. One library contains 12,000 strains that correspond to 4,583 ORFs in strain KPN1H1 with the KPC-3 carbapenemase gene deleted and the other library has approximately 4,570 mapped transposon insertion site in ATCC 43816, although the number of unique ORFs disrupted is unclear [61,62]. These libraries represent invaluable genetic resources that will not only advance our understanding *K*. *pneumoniae* pathogenesis and molecular biology within these specific strains, but can also be used as templates to generate insertional mutants in other strains or study the contribution of individual domains to phenotypes of interest. Furthermore, a small, condensed library, as described here, provides an invaluable tool for circumventing bottle necks during *in vivo* TnSeq studies.

In summary, we have generated a rich data set of mutants with a range of effects on CPS biosynthesis and hmv. These data provide a framework for future studies focused on identifying the precise signals that regulate CPS biosynthesis and hmv, as well as dissecting how these two major features of hvKp independently and coordinately impact pathogenesis. We have shown that CPS biosynthesis and hmv are coordinated processes that can be dissociated by deleting genes tied to central metabolism. The assembly of CPS and formation of hmv are energetically expensive processes, so it is intuitive that these processes are hardwired to the metabolic pulse of the cell. The linkage between hypervirulent and invasive *K*. *pneumoniae* and its overproduction of CPS and hmv may provide a fitness advantage for invasive infections, but at a metabolic cost. It is possible that in more stringent environments the metabolic burdens of elevated CPS biosynthesis and hypermucoviscosity may pose a fitness disadvantage. This cost-benefit balance between adequate energy sources and resisting environmental stresses, such as a healthy immune response or shear stress, may explain the emergence of the hvKp lineage and its invasive pathology compared to cKp strains.

## Materials and methods

### Ethics statement

All animal studies were conducted in accordance with the recommendations in the *Guide for the Care and Use of Laboratory Animals* [63]. The University of Michigan Institutional Animal Care and Use Committee (IACUC) approved this research (PRO00007474).

### Bacterial strains and media

*Klebsiella pneumoniae* strain KPPR1, a rifampin-resistant derivative of ATCC 43816, was used for all studies [29]. All primers, strains and plasmids described in these studies are detailed in **S1** and **S2 Tables.** Bacteria were cultured in lysogeny broth (LB) (5 g/L yeast extract, 10 g/L tryptone, 0.5 g/L NaCl) at 200 rpm and 37°C, unless otherwise noted. When appropriate, antibiotics were added at the following concentrations, rifampin (30 μg/mL), kanamycin (25 μg/mL), chloramphenicol (80 μg/mL), and spectinomycin (50 μg/mL). *Escherichia coli* strain TOP10 was used to generate complementation vectors and cultured in LB supplemented with chloramphenicol (20 μg/mL).

### Transposon library construction and sequencing

A library of random transposon mutants was generated in *K*. *pneumoniae* KPPR1 by conjugation with *E*. *coli* S17 harboring pSAM_Cam with a modified Mariner *Himar1* transposon as previously described [30]. Briefly, mid-log cultures of the donor and recipient strains were mixed in a 2:1 ratio, washed with PBS, resuspended in LB medium and spread on filter disks on top of an LB agar plate. Following a 2 hr incubation at 37°C, filters were transferred to an agar plate containing 250 μM IPTG (Invitrogen, Carlsbad, CA) and incubated for 2.5 hr at 37°C to induce expression of the transposase, enabling mobilization of the transposon. Bacteria were resuspended in LB medium transferred from the filter to LB agar with rifampin (30 μg/mL) and kanamycin (50 μg/mL) to select KPPR1 isolates with genomic transposon insertions. Rifampin-, kanamycin-resistant trans-conjugants were inoculated into 192, 96-well microplates containing 200 μL LB medium with 15% (v/v) glycerol and 50 μg/mL kanamycin and incubated statically at 37°C until saturation.

To verify that rifampin-, kanamycin-resistant colonies did not result from integration of the conjugation plasmid pSAM_Cam, a subset of the library was subjected to colony PCR utilizing primer pairs with homology to the plasmid backbone and the transposon as described previously (n = 10) [30]. The library was also tested to ensure that transposon mutants contained a single transposon insertion and that the insertion location was random by subjecting EcoRI-digested genomic DNA to Southern blotting using a probe homologous to the transposon as described previously (n = 13) [30].

Identification of the location of the transposon insertion site within the KPPR1 chromosome for each individual mutant was accomplished using next-generation sequencing coupled with Cartesian pooling to reduce the total number of samples to be sequenced. Using the method presented in [28] the number of samples to be sequenced is condensed first from 18,432 total mutants to 80 mutant pools representing the physical location of the mutants in the X, Y, and Z planes within each stack of 96, 96-well microplates. Each condensed pool of mutants is assigned a 6 bp barcode and the representation of each mutant within a barcoded pool is used to de-convolute the physical location within the library. To generate the mutant pools, transposon library plates were replicated into 75 μL of LB broth with kanamycin and cultured statically at 37°C overnight. The following day, 75 μL of 50% sterile glycerol was added to each plate, then Cartesian pooling was executed as previously described [28]. All intermediate mutant pools were stored at -20°C. Intermediate XY and Z pools were thawed and combined [28].

Genomic DNA was isolated from 1 mL of the combined final XY and Z pools using the DNeasy Blood and Tissue kit according to the manufacturer’s directions for gram-negative bacteria (Qiagen). Genomic DNA (1 μg) for each mutant pool was sheared using a Covaris DNA fragmentation system (Intensity = 5; duty cycle = 5%; cycles per burst = 200; 55 s), resulting in an average fragment size of 370 bp and ranging from 200–700 bp. Sheared DNA was blunt-end repaired and dA-Tailing was added using the NEBNext Ultra-End Repair/dA-Tailing Module. The DNA was then purified using AMPure XP, eluting in 25 μl water. All down-stream library preparation and sequencing data analysis was performed as described previously (**S1 Table**) [28,64].

### Condensed library construction

The KPPR1 genome (GCA_000742755.1) was used to identify predicted ORFs and gene coordinates and the fgenesB predictor was used to identify predicted transcriptional units [37]. The TP ID for each transposon insertion identified was manually matched to the plate number as described in [28]. The Fuzzy Join function of the Fuzzy Lookup Add-In for Microsoft Excel was then used to match the transposon insertion sites, plate (TP) and well (AP) coordinates, gene name, and gene coordinates. All Fuzzy Join functions had a similarity threshold = 1. The percent of the gene disrupted by the transposon insertion was calculated using the following two equations, where **Eq 1** was used for genes on the positive-strand and **Eq 2** was used for genes on the negative-strand:

$$\%\;of\;gene\;disrupted=100*\frac{transposon\;insertion\;site-gene\;start\;coordinates}{gene\;length}$$ (1)

$$\%\;of\;gene\;disrupted=100-\left(100*\frac{transposon\;insertion\;site-gene\;start\;coordinates}{gene\;length}\right)$$ (2)

Plate and well coordinates with multiple mutants mapped to the location were identified and counted using basic Excel functions.

The ordered library was then curated to identify optimal transposon mutants to be included in the condensed, ordered library. All intergenic mutations were removed from the data set and the resulting data set was sorted by gene name and then by percent of the gene disrupted. Fuzzy Join was then used to identify one transposon mutant for each gene in the KPPR1 genome. This condensed library was then evaluated and hand-curated to ensure that the positional location of the transposon mutant selected for the condensed library was identified with high confidence and contained a single insertion site, if possible. Selected transposon mutants were re-arrayed into microplates containing LB/kanamycin medium, grown statically overnight at 37°C, mixed with an equal volume of 50% glycerol then stored at -80°C to make the condensed, ordered library.

### Hypermucoviscosity sedimentation assays

The hmv was assessed as described previously with the following modifications [30]. The overnight cultures were pelleted at 21,000 x *g* for 15 min then resuspended to an OD_600_ = 1.0 in a final volume of 1 mL PBS. Samples were centrifuged at 1,000 x *g* for 5 min and the OD_600_ of the upper 900 μL supernatant was determined in a 1 cm cuvette.

### Uronic acid quantification

Analysis of the total uronic acid content was performed following a modified procedure [40]. A 0.25 mL volume of overnight culture was mixed with 50 μL 1% Zwittergent 3–14 in 100 mM citric acid buffer, pH 2 at 50°C for 20 min. Bacterial cells were pelleted by centrifugation then 0.1 mL of the cell-free supernatant was mixed with 0.4 mL absolute ethanol and incubated according to [40]. Samples were rehydrated in 0.2 mL of water then 1.2 mL of 0.0125 M sodium tetraborate in concentrated sulfuric acid was added. All subsequent steps were as described in [40] and normalized to the total OD_600_.

### Forward screen

Microplates containing the condensed, ordered library (total of 3,733 mutants) were thawed at room temperature and replicated into 100 μL of LB in round bottom microplates. Plates were wrapped with plastic wrap to prevent evaporation and incubated statically at 37°C for 18–19 h. The sedimentation assay was adapted to a microplate format as follows. Plates were vortexed on low for 60 sec then the total OD_600_ was recorded. Plates were centrifuged at 2,000 x *g* for 20 min, then the upper 50 μL of supernatant was transferred to a new microplate to measure the OD_600_. Transposon mutants with a total OD_600_ less than two standard deviations from the plate mean and a supernatant OD_600_ more than two standard deviations from the plate mean were considered hits. The hits were struck onto LB agar, incubated at 37°C overnight and evaluated by string test the following day. Three colonies of each transposon mutant confirmed as non-mucoviscous or hypo-mucoviscous by string test were arrayed into a microplate for confirmation. The same work-flow with sedimentation and string test were repeated with the arrayed hits for confirmation. The top hits were confirmed in a third sedimentation assay where the transposon mutants were cultured in 3 mL of LB medium overnight at 37°C with aeration, then the OD_600_ of 100 μL of the total culture and culture supernatant was determined in a microplate before and after centrifugation at 7,000 x *g* for 10 min. Pathway analysis was performed using KEGG GENES (Kyoto Encyclopedia of Genes and Genomes) [35,36].

### Reverse screen

Transposon mutants were revived on LB agar plates, then individual colonies were inoculated into 3 mL of LB medium and incubated overnight at 37°C with aeration. Uronic acid quantification was performed as described above in parallel with a modified sedimentation assay. The sedimentation assay was performed by recording the OD_600_ of 100 μL of overnight culture in a microplate, followed by pelleting 1 mL of the overnight culture at 7,000 x *g* for 10 min, and quantifying the OD_600_ of the upper 100 μL of the culture. The ratio of supernatant to total OD_600_ was used as a measure of hmv.

### Transmission electron microscopy

A pellet of cells was resuspended in fixative (2.5% glutaraldehyde, 1.25% paraformaldehyde and 0.03% tannic acid in 0.1 M cacodylate buffer, pH 7.2) for at least 2 h at 4°C. The cells were centrifuged at 21,000 x *g* for 5 m and the fixative removed. Fixed cells were embedded in 4% agarose, Type I-A (Sigma) and the agarose button stored in 200 μL of fixative and stored at 4°C overnight. Afterwards, all sample preparation was performed at room temperature unless otherwise noted. Samples were washed in 0.1 M cacodylate buffer and post-fixed with 2% osmium tetroxide/1.5% potassium ferrocyanide in 0.1 M cacodylate buffer for 1 h, washed three times in 0.1 M cacodylate buffer, three times in 0.1 M sodium acetate buffer (pH 5.2), stained in 2% uranyl acetate in 0.1 M sodium acetate buffer for 1 h, washed twice in 0.1 M sodium acetate buffer and once in water, then dehydrated in grades of ethanol (4°C, 15 m each: 30%, 50%, 70%, 80%, 90%, 95%) then twice in 100% ethanol at room temperature and once in 100% acetone (each 15 m). Samples were infiltrated with acetone:Spurr’s resin (2:1) for 1 h, 1:1 for 2 hr, 1:2 for 16 h, then absolute Spurr’s resin for 24 h. Samples were embedded for 30 m and polymerized at 65°C for 24 h. Samples were sectioned at 70 nm thickness (Leica UC7 ultra microtome), put on 200 mesh copper grids and post-stained with 4% uranyl acetate followed by Reynolds’ lead citrate, and imaged with a JEOL 1400 Transmission Electron Microscope using an AMT XR 401 camera.

### Construction and complementation of mutants

Insertional mutants were generated using λ Red recombineering adapted to *K*. *pneumoniae* as described previously with the following exceptions [30,65]. All bacterial cultures for competent cells were supplemented with 0.5 μM EDTA, which improves centrifugation. Electrocompetent cells were either transformed immediately or flash frozen and stored at -80°C for future use. PCR products with 60 base pairs of homology flanking the region targeted for deletion were digested with DpnI and 6 μL of the column purified PCR product was mixed with electrocompetent KPPR1 pKD46 cells and incubated on ice for 30 min. Cells were electroporated in a 0.1-cm-gap cuvette at 1.8 kV, 400 Ω, and 25 μF [66]. Transformants were recovered with 500 μL of LB and static incubation at room temperature overnight, although some mutants required recovery at 30°C for 3–4 hr or 37°C for 1–2 h, with shaking.

All mutants were generated using pKD4 template, which confers kanamycin resistance. Successful mutagenesis was confirmed by PCR and restriction digest with EagI. All oligonucleotides for mutagenesis and confirmation are listed in **S1 Table**. Mixed colony morphology was observed when Δw*za* and Δ*aceE* strains were cultured without kanamycin, so these strains were maintained with kanamycin (25 μg/ml) as long as experimental conditions permitted.

Complementation vectors were generated using NEBuilder HiFi DNA Assembly Cloning Kit (New England BioLabs). Primers were designed using the online NEBuilder Assembly Tool with the following settings: >20 nucleotide overlap, Phusion DNA Polymerase (HF Buffer), 500 nM primer concentration (**S1 Table**). The ORF and 500 bp of the predicted promoter region were exchanged with 600 bp of the *tet* cassette or 200 bp of the *cat* cassette in pACYC184 [67]. Gel purified PCR products were assembled according to the manufacturer’s instructions and the enzymatic reaction was incubated at 50°C for 1 h. The NEBuilder reaction was dialyzed overnight against 10% sterile glycerol using a VSWF 0.025 μm filter disk. The dialyzed DNA was collected and electroporated into *E*. *coli* TOP10 cells. pACYC184Δ*tet* and pACYC184Δ*cat* vectors were generated by ligating the pACYC184 PCR product without an insert, effectively eliminating 600 bp of the *tet* or 200 bp of the *cat* cassette. The resulting plasmids were verified by restriction digest and Sanger sequencing then 0.5 μL of DNA was transformed into 50 μL of electrocompetent *K*. *pneumoniae* mutants [30].

### Cell culture and association assays

A549 cells (ATCC CCL185) derived from a human lung carcinoma were maintained in Ham’s F-12K (Kaighn’s) medium (Gibco) supplemented with 10% heat-inactivated fetal calf serum (Corning), 100 U/ml penicillin, and 100 μg/ml streptomycin in an atmosphere of 5% CO_2_.

Bacterial pellets from stationary phase cultures were resuspended in 1 mL F-12K without additives then normalized to 2x10^7^ CFU/ml (OD 0.02). Confluent A549 cells (~5x10^5^ cells/well) in 24-well tissue culture dishes were washed with 1 mL of PBS then 1 mL of 2x10^7^ CFU/ml bacteria (MOI 50) in additive-free F-12K were added to each well. Samples were spun at 500 rpm (54 x *g*) for 5 min then incubated at 37°C, 5% CO_2_ for 1 h, followed by an incubation at 4°C for 1 h. Samples were washed three times with PBS then lysed with 1 mL of 0.2% Triton-X100 in PBS for 5–10 min. Input and cell-associated bacterial counts were determined by serial dilution and CFU enumeration.

### Serum survival

Pooled human complement serum (Innovative Research) was divided into 1 mL aliquots, half the samples were heat-inactivated at 56–58°C for 1 h, and all were flash-frozen and stored at -80°C. Bacterial pellets from stationary phase cultures were resuspended in 1 mL PBS then normalized to 2x10^6^ CFU/ml. 10 μL of the bacterial suspension was added to 90 μL of active or heat-inactivated human serum then gently vortexed. Plates were sealed with plastic wrap and incubated at 37°C for 90 min. Input and output bacterial counts were determined by serial dilution and CFU enumeration.

### *In vitro g*rowth analyses

#### Mono-culture growth

Bacterial strains were cultured statically overnight in triplicate in 100 μL of LB medium in a microplate at 37°C. The cultures were normalized to an OD_600_ of 0.01 in LB medium then 100 μL was aliquoted into a microplate. A Bioscreen-C Automated Growth Curve Analysis System (Growth Curves USA) was used to record the OD_600_ every 15 min for 24 h. Cultures were incubated at 37°C with continuous, medium shaking. The doubling time was determined by identifying two time points (t_2_ and OD_2_ = late time point and t_1_ and OD_1_ = early time point) within the logarithmic growth phase, then applying **Eq 3**:

$$\mathrm{doubling}\;\mathrm{time}\;(\min)=60\times [\ln\left(2\right)÷\ln\;\left(\frac{OD_{2}/OD_{1}}{t_{2}-t_{1}}\right)]$$ (3)

#### Competitive growth

Bacteria were grown as described for mono-culture assays, with the following modifications. Bacterial pellets from overnight cultures were washed once in PBS then diluted to 1x10^8^ CFU/ml in PBS. Each mutant strain was mixed 1:1 with WT and 5 μL of the 1:1 mixture was added to 45 μL of growth medium (LB or BALF) in a Bioscreen-C plate. After 24 h of growth, the output ratio of WT:mutant was determined. Input and output ratios were determined by serial diluting and spot plating on LB+rif and LB+rif+kan plates. BALF was collected from 6–8 week/old C57Bl/6 mice and prepared as described previously [68].

### Murine pneumonia model

A murine model of *K*. *pneumoniae* infection was used as previously described [30]. Briefly, 6–8 week/old C57BL/6 mice (Jackson Laboratory, Bar Harbor, ME) were anaesthetized with isoflurane and retropharyngeally inoculated with 1 x 10^6^ CFU *K*. *pneumoniae* in 50 μL of PBS. All bacterial strains were cultured overnight in 50 mL LB. Bacteria were pelleted at 10,000 x *g* for 30 min and the pellets resuspended in sterile PBS to a final OD_600_ of 2.0. WT and mutant were mixed at a 1:1 ratio and the input colony forming units (CFU) ratios determined by serial dilution and drip plating on both LB and LB+kan. Infections were allowed to proceed for 24 hr and mice were euthanized by CO_2_ asphyxiation. Blood was collected by cardiac puncture in heparinized tubes. Lungs and spleens were collected and homogenized in 3 mL of sterile PBS. Whole blood and homogenized lungs and spleens were serial diluted in PBS and 10 μL drip plated on LB and LB+kan. Plates were incubated at 30°C overnight and the CFUs enumerated the following morning. The limit of detection was 100 CFU/mL and all samples without detectable CFU counts were analyzed assuming that they contained 99 CFU/mL. The competitive index (CI) was calculated as in **Eq 4**.

$$CI=\frac{\left(output\;mutant\;CFU/mL\right)/\left(output\;WT\;CFU/mL\right)}{\left(input\;mutant\;CFU/mL\right)/\left(input\;WT\;CFU/mL\right)}$$ (4)

### Statistical analysis

All *in vitro* replicates represent biological replicates and all *in vivo* studies were replicated at least twice. All statistical analyses were computed in Prism 8.3.0 (GraphPad Software, La Jolla, CA). For *in vitro* experiments, significance was calculated using unpaired *t*-tests and the Holm-Sidak method to correct for multiple comparisons with alpha = 0.05. A two-tailed P value for the correlation between hmv and CPS production was computed by nonparametric Spearman correlation with a 95% confidence interval. For competitive growth experiments, all competitive indices were log_10_ transformed then significance was calculated using a one sample *t* test, where the actual mean was compared to a theoretical mean of 0.00 (no fitness defect). Results were considered significant if the P value was less than or equal to 0.05.

## Supporting information

S1 Fig The centrifugation assay recapitulates the string test results of forward screen hits. Hits in the forward screen were categorized as (A) hypo-mucoid (hmv^low^) or (B) non-mucoid (hmv^0^) by string test and sedimentation assays performed in microplates. To confirm that categorizing mutants based on these high-throughput methods is reflected in full-scale centrifugation assays, 3 mL of each mutant was grown overnight and centrifuged at 7,000 x *g* for 10 min. The optical density at 600 nm (OD_600_) of the supernatant was normalized to the total OD_600_ of the overnight culture by measuring the absorbance of 100 μL in a plate reader. Error bars represent one standard deviation from the mean of the assay performed in triplicate. Statistical significance between wildtype (WT) and each mutant was determined using the Holm-Sidak method, with alpha = 0.05. Computations assumed that all rows were sampled from populations with the same scatter. No results were significantly different from WT.
(TIF)

S2 Fig Validation of key mutants. The capsular polysaccharide (CPS) production and hypermucoviscosity (hmv) of select targeted deletion mutants were examined by (A, C, E) uronic acid quantification and (B, D, F) sedimentation (1 OD_600_ unit centrifuged at 1,000 x *g* for 5 min), respectively. (A-D) Strains were transformed with either vector alone or a complementation vector, which contained the targeted gene under the control of its native promoter. The vector backbone was (A-B) pACYC184Δ*tet* or (C-D) pACYC184Δ*cat*. (E-F) Five independently generated isogenic Δ*sdhA* mutants (isolates #1, 2, 7, 9, and 11) were evaluated. All error bars represent the standard error of the mean and each assay was performed at least three times, each with biological triplicates. Statistical significance between wildtype (WT) and each mutant was determined using unpaired t-tests and the Holm-Sidak method to correct for multiple comparisons, with alpha = 0.05. Computations assumed that all data points were sampled from populations with the same scatter where, * P < 0.05; ** P < 0.01; *** P < 0.001; # P < 0.0001.
(TIF)

S3 Fig Absolute bacterial counts from competitive infections. C57Bl/6 mice were infected retropharyngeally with 1 x 10^6^ colony forming units (CFU) of a 1:1 ratio of wildtype (WT) to mutant. After 24 h of infection, the bacterial burdens of WT and each mutant in the (A) lungs, (B) blood, and (C) spleens were determined by differential plating. Each dot represents an individual mouse and the limit of detection (LOD) was 297 CFU/organ. All CFU counts were log_10_-transformed.
(TIF)

S4 Fig *In vitro* growth of key mutants. The *in vitro* growth of the six mutants co-inoculated with wildtype (WT) in a murine model of pneumonia was evaluated. Mutants were grown in LB and growth quantified by (A and B [inset of A]) measuring the optical density at 600 nm (OD_600_) each hour (hr) and (C) integrating the area under the growth curve in GraphPad Prism 8.3.0. Error bars represent the standard error of the mean and each data point represents at least nine replicates. In some instances, error bars are plotted, but not visible. Statistical significance between the area under the curve and doubling time of wildtype (WT) and each mutant was determined using the Holm-Sidak method, with alpha = 0.05. Computations assumed that all data points were sampled from populations with the same scatter where, * P < 0.05; ** P < 0.01; *** P < 0.001; # P < 0.0001.
(TIF)

S1 Table Primers used in this study. (XLSX)

S2 Table Strains and plasmids used in this study. ^a^Km = kanamycin; Cm = chloramphenicol; Tc = tetracycline; Rif = rifampin; Sp = spectinomycin.
(XLSX)

S1 Data Transposon insertion sites and positional locations within the full and condensed ordered libraries. (Tab 1) Legend for this data set. The nucleotide and gene location for each transposon insertion is reported in conjunction with the positional location and confidence with which that location was mapped for the full library. Transposons are reported based on if they (Tab 2) disrupt a single gene, (Tab 3) are intergenic, and (Tab 4) disrupt two genes. (Tab 5) All remaining genes not disrupted in the full library. (Tab 6) Map decoding the TP ID with the plate location for the full, ordered library. (Tab 7) Transposon mutants and their positional locations in the condensed library. All genes reported in this study are annotated using the old locus tags. (Tab 8) The old locus tag, its nucleotide location, and gene function have been matched with the new locus tags.
(XLSX)
