# Supplementary figures and images for: A systematic analysis of hypermucoviscosity and capsule reveals distinct and overlapping genes that impact Klebsiella pneumoniae fitness
Source: PLoS Pathog. 2021 Mar 15;17(3):e1009376. doi: 10.1371/journal.ppat.1009376 (PMC7993769; doi:10.1371/journal.ppat.1009376)

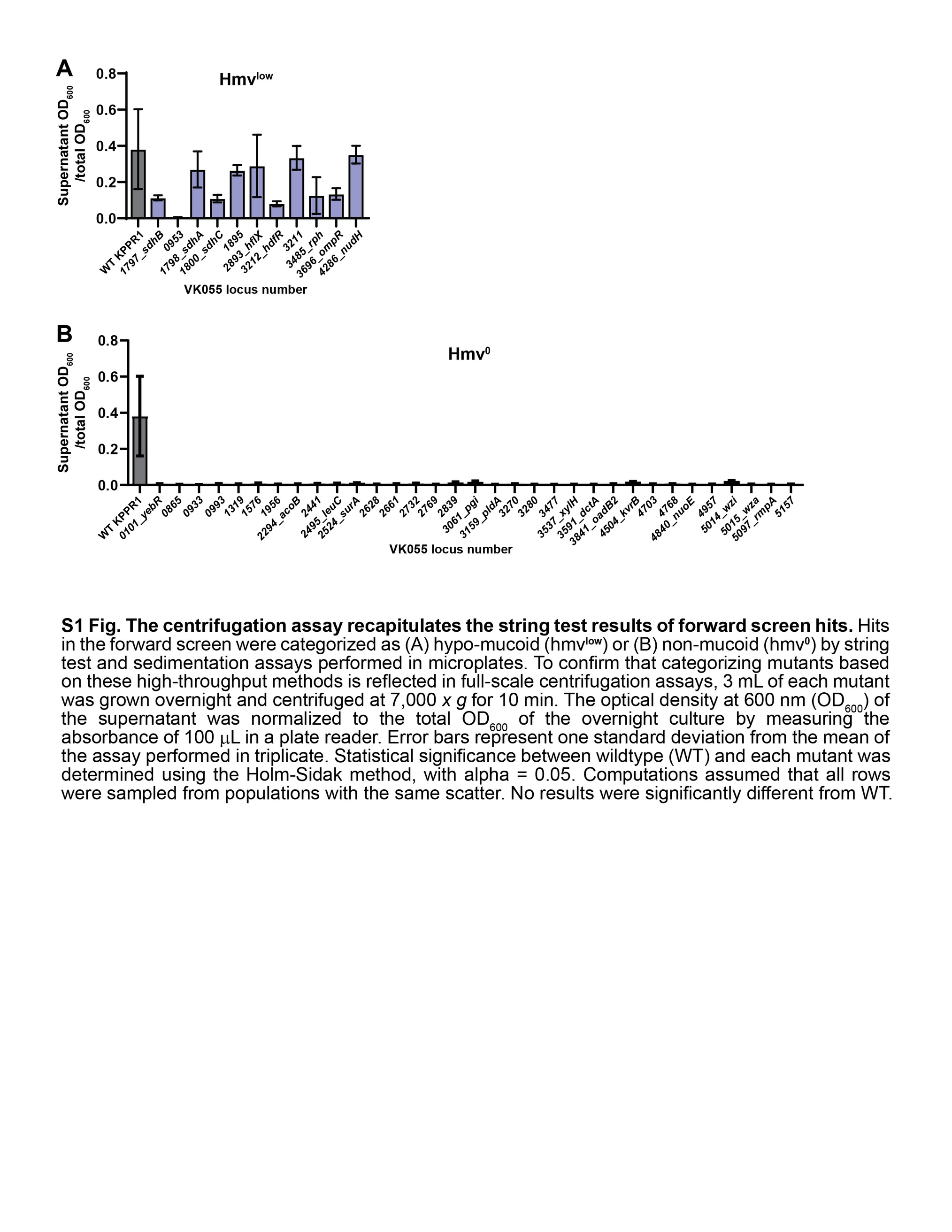

Supplement: S1 Fig — Hits in the forward screen were categorized as (A) hypo-mucoid (hmvlow) or (B) non-mucoid (hmv0) by string test and sedimentation assays performed in microplates. To confirm that categorizing mutants based on these high-throughput methods is reflected in full-scale centrifugation assays, 3 mL of each mutant was grown overnight and centrifuged at 7,000 x g for 10 min. The optical density at 600 nm (OD600) of the supernatant was normalized to the total OD600 of the overnight culture by measuring the absorbance of 100 μL in a plate reader. Error bars represent one standard deviation from the mean of the assay performed in triplicate. Statistical significance between wildtype (WT) and each mutant was determined using the Holm-Sidak method, with alpha = 0.05. Computations assumed that all rows were sampled from populations with the same scatter. No results were significantly different from WT. (TIF) [file ppat.1009376.s001.tif]

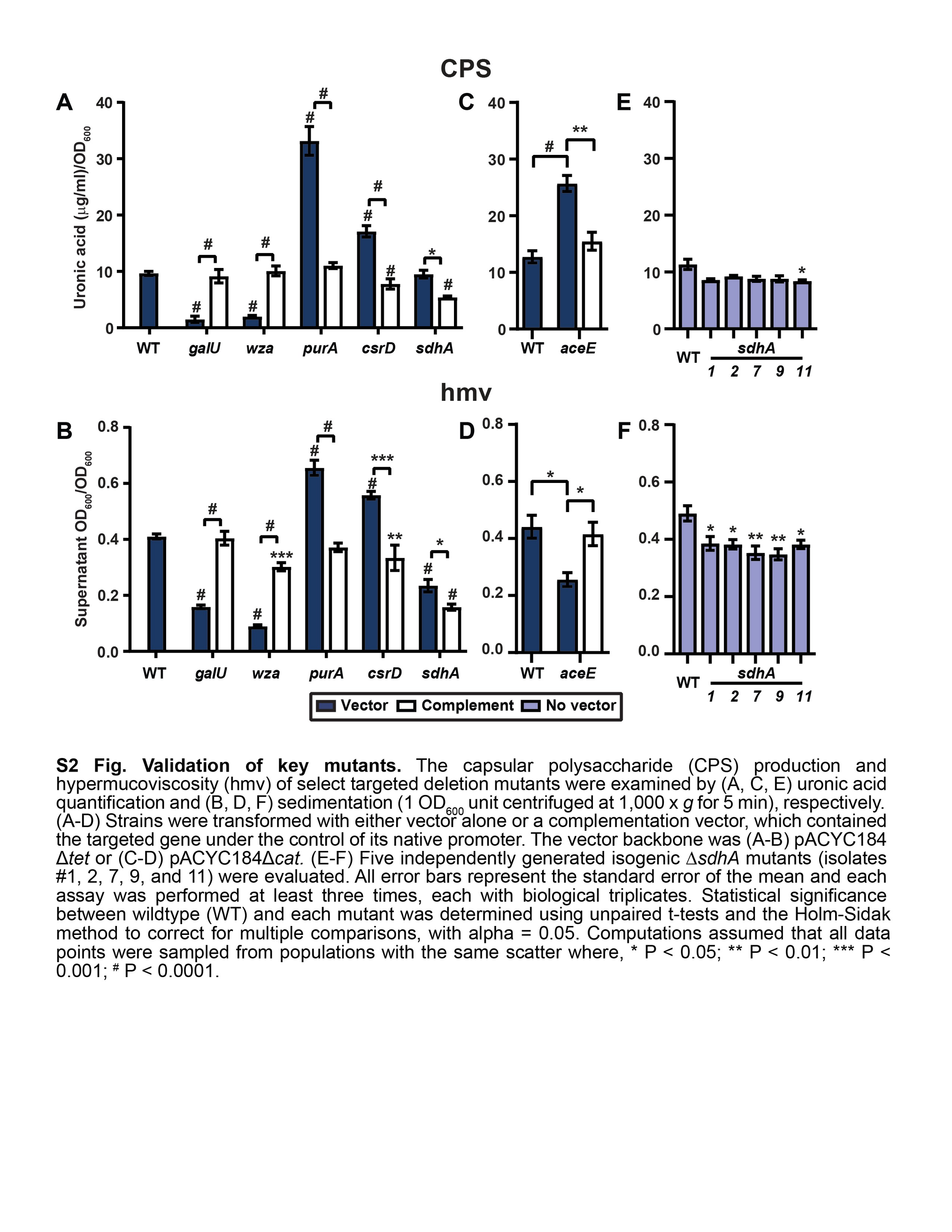

Supplement: S2 Fig — The capsular polysaccharide (CPS) production and hypermucoviscosity (hmv) of select targeted deletion mutants were examined by (A, C, E) uronic acid quantification and (B, D, F) sedimentation (1 OD600 unit centrifuged at 1,000 x g for 5 min), respectively. (A-D) Strains were transformed with either vector alone or a complementation vector, which contained the targeted gene under the control of its native promoter. The vector backbone was (A-B) pACYC184Δtet or (C-D) pACYC184Δcat. (E-F) Five independently generated isogenic ΔsdhA mutants (isolates #1, 2, 7, 9, and 11) were evaluated. All error bars represent the standard error of the mean and each assay was performed at least three times, each with biological triplicates. Statistical significance between wildtype (WT) and each mutant was determined using unpaired t-tests and the Holm-Sidak method to correct for multiple comparisons, with alpha = 0.05. Computations assumed that all data points were sampled from populations with the same scatter where, * P < 0.05; ** P < 0.01; *** P < 0.001; # P < 0.0001. (TIF) [file ppat.1009376.s002.tif]

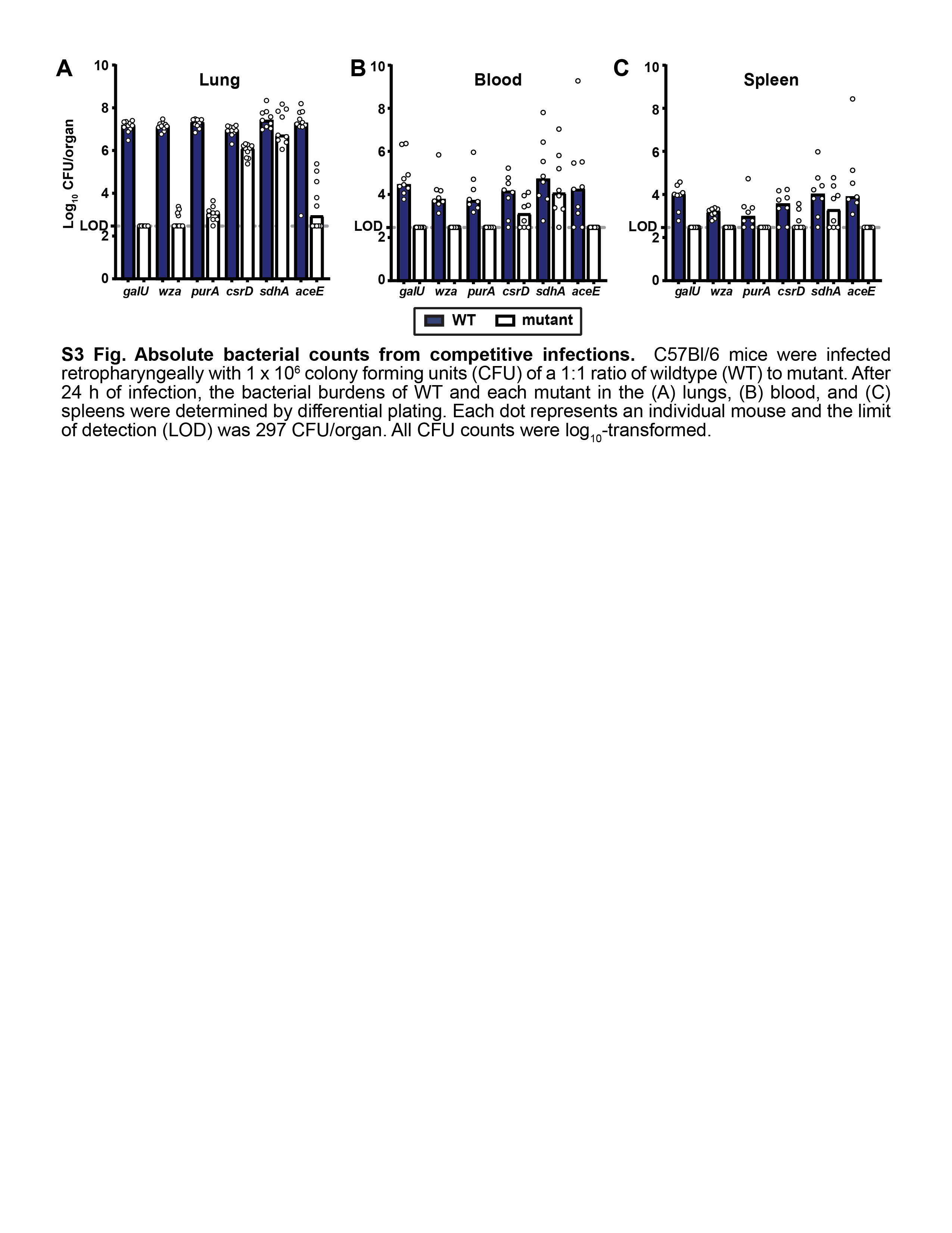

Supplement: S3 Fig — C57Bl/6 mice were infected retropharyngeally with 1 x 10^6^ colony forming units (CFU) of a 1:1 ratio of wildtype (WT) to mutant. After 24 h of infection, the bacterial burdens of WT and each mutant in the (A) lungs, (B) blood, and (C) spleens were determined by differential plating. Each dot represents an individual mouse and the limit of detection (LOD) was 297 CFU/organ. All CFU counts were log10-transformed. (TIF) [file ppat.1009376.s003.tif]

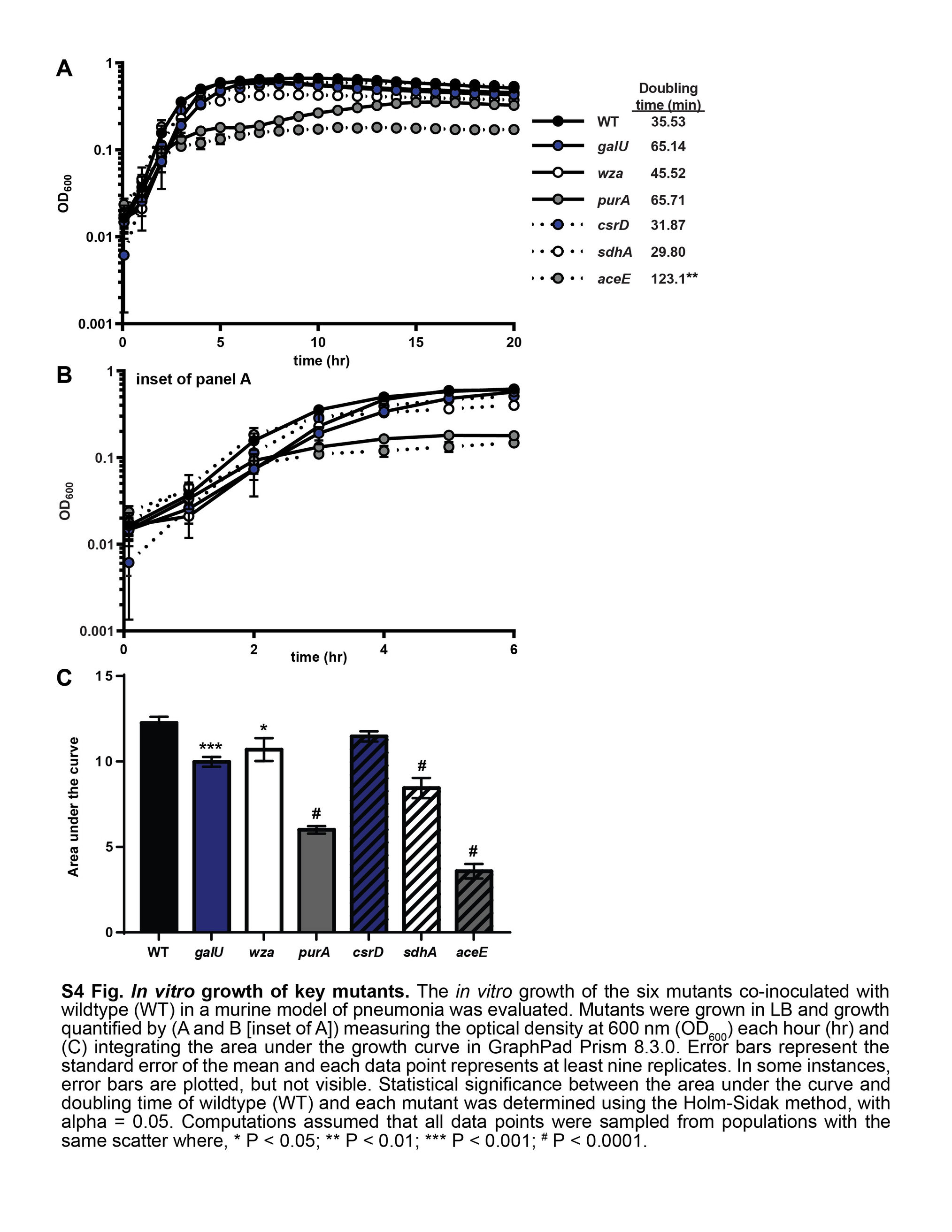

Supplement: S4 Fig — The in vitro growth of the six mutants co-inoculated with wildtype (WT) in a murine model of pneumonia was evaluated. Mutants were grown in LB and growth quantified by (A and B [inset of A]) measuring the optical density at 600 nm (OD600) each hour (hr) and (C) integrating the area under the growth curve in GraphPad Prism 8.3.0. Error bars represent the standard error of the mean and each data point represents at least nine replicates. In some instances, error bars are plotted, but not visible. Statistical significance between the area under the curve and doubling time of wildtype (WT) and each mutant was determined using the Holm-Sidak method, with alpha = 0.05. Computations assumed that all data points were sampled from populations with the same scatter where, * P < 0.05; ** P < 0.01; *** P < 0.001; # P < 0.0001. (TIF) [file ppat.1009376.s004.tif]
